# Circadian regulation of muscle growth independent of locomotor activity

**DOI:** 10.1073/pnas.2012450117

**Published:** 2020-11-23

**Authors:** Jeffrey J. Kelu, Tapan G. Pipalia, Simon M. Hughes

**Affiliations:** ^a^Randall Centre for Cell and Molecular Biophysics, King’s College London, London SE1 1UL, United Kingdom

**Keywords:** muscle, zebrafish, circadian rhythm

## Abstract

Muscle tissue shows diurnal variations in function, physiology, and metabolism. Whether such variations are dependent on the circadian clock per se or are secondary to circadian differences in physical activity and feeding pattern is unclear. By measuring muscle growth over 12-h periods in live prefeeding larval zebrafish, we show that muscle grows more during day than night. Expression of dominant negative CLOCK (ΔCLK), which inhibits molecular clock function, ablates circadian differences and reduces muscle growth. Inhibition of muscle contraction reduces growth in both day and night, but does not ablate the day/night difference. The circadian clock and physical activity are both required to promote higher muscle protein synthesis during the day compared to night, whereas markers of protein degradation, *murf* messenger RNAs, are higher at night. Proteasomal inhibitors increase muscle growth at night, irrespective of physical activity, but have no effect during the day. Although physical activity enhances TORC1 activity, and the TORC1 inhibitor rapamycin inhibits clock-driven daytime growth, no effect on muscle growth at night was detected. Importantly, day/night differences in 1) muscle growth, 2) protein synthesis, and 3) *murf* expression all persist in entrained larvae under free-running constant conditions, indicating circadian drive. Removal of circadian input by exposure to either permanent darkness or light leads to suboptimal muscle growth. We conclude that diurnal variations in muscle growth and metabolism are a circadian property that is independent of, but augmented by, physical activity, at least during development.

## Significance

A long-standing question, particularly in physiotherapy and sports medicine, is whether time of day affects muscle metabolism and hence growth, either intrinsically or in response to exercise or nutrition. Answers would help to identify the best time of day to exercise, build muscle, and prevent aging- or disease-related sarcopenia. Here, we address this question in live zebrafish myotome in vivo, without interference from other circadian oscillations such as locomotor activity and food intake. We show that active muscle anabolizes more in the day and grows faster, while catabolizing more at night and growing slower. Such day/night differences remain in inactive muscle but disappear after clock disruption. We conclude that muscles display circadian differences in growth independent of activity and feeding.

Animals exhibit circadian rhythms. An obvious example is the diurnal/nocturnal pattern of locomotor activity, which is powered by contraction of skeletal muscle. Diurnal variations are also observed in muscle performance and muscle physiology (1–3), but whether they represent a circadian paradigm requires critical evaluation. For example, to be considered truly circadian, rhythms have to persist under constant conditions, that is, in the absence of zeitgebers (environmental entrainment cues) (4). Studies in mammals demonstrated that skeletal muscle possesses hundreds to thousands of rhythmic genes and metabolites that are involved in metabolism, transcription, and signaling (5–11). The function of the circadian clock in these diurnal oscillations and their effects on muscle growth, however, remains largely elusive. Murine whole-body knockout of the core clock gene *Bmal1* results in severe defects in growth and muscle function (12, 13), but muscle-specific *Bmal1* knockout failed to recapitulate these phenotypes (14, 15), suggesting they might be secondary to clock disruption in other tissues. Indeed, central nervous system-driven rhythmic locomotor and feeding activity, which are well-established determinants of muscle growth, are lost in global *Bmal1* knockout (16, 17), but unaltered in muscle-specific *Bmal1* knockout (5, 15). However, muscle-specific *Bmal1* knockouts have yielded mild and variable results (15, 18). Thus, whether the circadian clock regulates muscle growth intrinsically and independent of altered physical activity is unclear. We recently developed a method of measuring muscle growth over a period of a few hours in the live zebrafish (19, 20). Here we use this zebrafish model to study the circadian regulation of muscle growth.

Zebrafish offer several advantages for the study of muscle circadian clock. First, zebrafish larvae are transparent, and all cells are directly entrainable by light, and therefore do not require a central oscillator (e.g., the superchiasmatic nucleus or pineal gland) for entrainment (21). Nevertheless, zebrafish have a central oscillator similar to that in other animals that may synchronize the oscillation of peripheral clocks in some circumstances (22, 23). Second, zebrafish require a zeitgeber such as light during early embryogenesis to entrain their clocks (23–25), so larvae lacking circadian rhythm can be produced. Third, muscle growth can be studied in anesthetized zebrafish in the absence of the confounding effect of locomotor activity (26). Fourth, zebrafish larvae do not feed until 5 d postfertilization (5 dpf), so the other prominent confounding effect, that of feeding, can be eliminated. Hence, zebrafish larvae enable study of the role of the circadian clock, independent of exercise and feeding, which has not been possible in other model systems.

Here we show that, in a 12-h light/12-h dark (LD) regime, muscle grows more during day than night. After removal of zeitgebers and/or physical activity, muscle growth continues to show circadian growth variations. Physical activity augments muscle growth in both light and dark phases. Our results show that muscle anabolism is greatest in daytime, is promoted by physical activity, and correlates imperfectly with TORC1 activity. Rapamycin, a TORC1 inhibitor, specifically reduced muscle growth in the day. In contrast, catabolism is greatest at night and correlates with accumulation of *murf* messenger RNAs (mRNAs), and proteasome inhibitors specifically enhanced growth at night. We show that muscle grows less in permanent darkness (DD) or light (LL) when a whole-body−synchronized circadian rhythm is never established, compared with LD, further arguing against an acute effect of light in stimulating muscle growth/anabolism during daytime. Importantly, we show that, after entrainment, diurnal variations in muscle growth and protein turnover persisted under constant conditions. This strongly suggests a circadian origin of the day/night variations. Expression of a dominant negative CLOCK (ΔCLK) disrupted molecular clock function, abolished circadian differences, and reduced muscle growth. We conclude that muscle metabolism and growth are directly regulated by the circadian clock independent of physical activity and food intake.

## Results

### Consistent Myotome Growth Despite Individual Variation in Size.

**Fig. 1.**
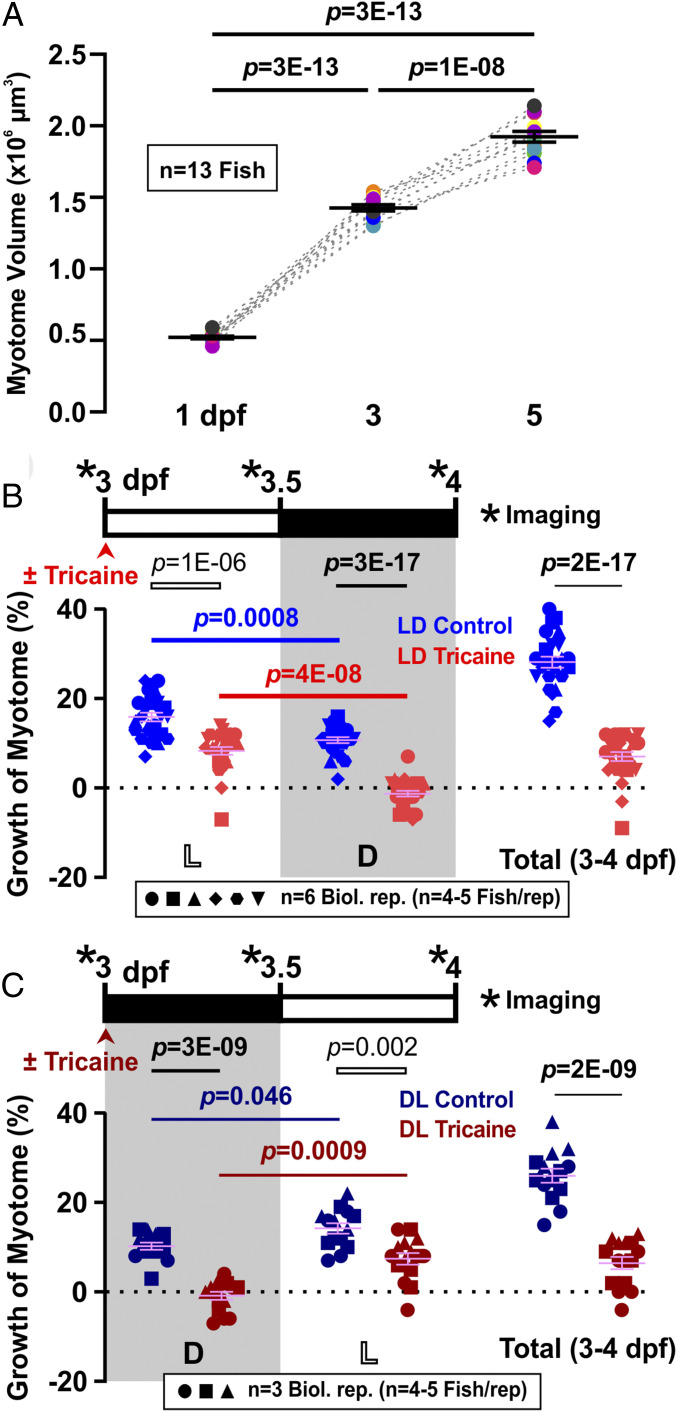
Diurnal variation and requirement for activity in volumetric growth of the myotome. (*A*) Growth of myotome in somite 17 between 1 and 5 dpf. Black bars represent mean ± SEM. Colors represent different individual fish from a single lay (*n* = 13 fish). (*B* and *C*) Larvae raised under LD (*B*; *n* = 25 or 26 fish from six biological replicates) or DL (*C*; *n* = 14 or 15 fish from three biological replicates) were either untreated (Control, blue/dark blue) or anesthetized (Tricaine, red/dark red) from 3 dpf to 4 dpf. Confocal imaging was performed every 12 h (*) over the 24-h period to measure myotome volume. Graphs show growth of myotome (percent) of individual fish over each 12-h period (left and center) or the full 24 h (right). Complete data on the same LD fish are shown in *SI Appendix*, Fig. S2 *A* and *B*. Symbol shapes distinguish biological replicates from separate lays. L, light; D, dark.

The volume of the zebrafish myotome of somite 17 grows by around 174% (0.9 × 10^6^ µm^3^) between 1 and 3 dpf, and a further 35% (0.5 × 10^6^ µm^3^) between 3 and 5 dpf (Fig. 1*A*). We select somite 17 because of its ease of imaging at the trunk−tail boundary (19, 20) (*SI Appendix*, Fig. S1*A*). Fiber numbers increase in parallel, but the major component of growth is increase in fiber size (*SI Appendix*, Fig. S1*B*). Individuals vary in myotome volume both within and between lays (*SI Appendix*, Fig. S1 *C*–*E*). When single unmanipulated fish were measured repeatedly at 3 and 4 dpf, all grew by a similar amount, although final size correlated with initial size (*SI Appendix*, Fig. S1*D*). We conclude that myotome volume can be accurately measured, that comparisons between individuals are best made within lays, and that growth can be most accurately assessed by repeatedly measuring single individuals.

### Muscle Grows More in Day than Night in a Light/Dark Cycle.

To assess day/night effects, fish were raised in an LD regime, and the growth of the myotome in posthatch larvae aged 3 dpf, a stage before independent feeding, was measured every 12 h over a 24-h period. The myotome grew about 50% faster during day than night, whether measured in absolute or percentage terms (Fig. 1*B* and *SI Appendix*, Fig. S2 *A* and *B*), and the growth difference was consistent across different lays and experiments (*SI Appendix*, Fig. S2*B*). This day/night variation correlates with the rhythmic expression of the core clock gene *bmal1a* between 3 and 5 dpf (*SI Appendix*, Fig. S2*C*). Interestingly, it appears that yolk consumption varies with size of individual larvae, but is constant over the diurnal cycle (*SI Appendix*, Fig. S3), arguing that altered supply of nutrients does not account for day/night variation in myotome growth. Measurement of growth over a 48-h period between 3 and 5 dpf consistently shows a growth difference between day and night, although overall growth is slower on day 4 than day 3 (*SI Appendix*, Fig. S2*D*). To distinguish whether the slower growth at night simply reflects the slowing of growth with developmental stage, the light cycle was reversed on sibling larvae (DL), and their myotome growth was measured (Fig. 1*C*). The larval myotome consistently grew more in the light (3.5 dpf to 4 dpf) than dark (3 dpf to 3.5 dpf) phase. We conclude that the zebrafish myotome grows more in the day than in the night, irrespective of developmental stage.

### Day/Night Differences in Growth Independent of Activity.

Diurnal zebrafish larvae spontaneously swim more during the day (27, 28), raising the possibility that activity promotes muscle growth in daytime. We therefore grew larvae in the anesthetic tricaine, which completely inhibited all movement, from 3 dpf to 4 dpf and measured myotome growth (Fig. 1*B* and *SI Appendix*, Fig. S2 *A* and *B*). We have previously shown that tricaine blocks movement by specifically inhibiting neural activity (29). Growth was significantly reduced by inactivity, during both day and night. However, a clear day/night growth difference remained (Fig. 1*B* and *SI Appendix*, Fig. S2 *A* and *B*), although the clock appears to be somewhat delayed by anesthesia (*SI Appendix*, Fig. S2*C*). The myotome of inactive fish grew during the day but failed to grow, and even showed a tendency to shrink, at night (Fig. 1*B*). Importantly, such day/night difference was shown to remain consistent over a 48-h period in anesthetized fish (*SI Appendix*, Fig. S2*D*). When the light cycle was reversed, again, the myotome of anesthetized fish grew in the later light phase and ceased growing in the earlier dark phase (Fig. 1*C*). These data show that day/night differences in growth are independent of muscle electrical and contractile activity. Interestingly, the reduction in growth caused by inactivity did not appear different between day and night (Fig. 1 *B* and *C*), suggesting that the amount of activity-dependent growth is not proportional to the quantity of activity per se.

### Physical Activity and Daytime Promote Anabolism.

**Fig. 2.**
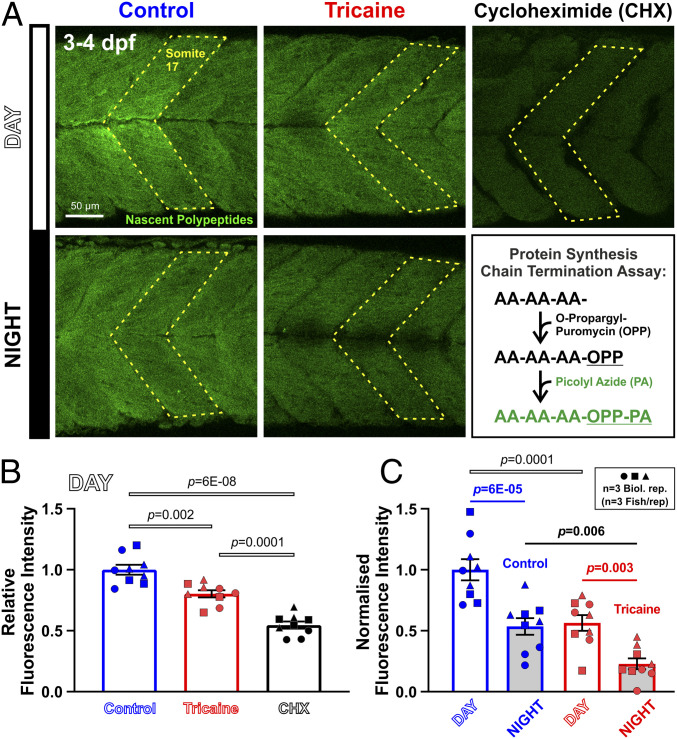
Diurnal variation and effect of activity in muscle protein synthesis. Larvae raised under LD that were either untreated (Control, blue) or anesthetized (Tricaine, red) at 3 dpf, and then treated with OPP for 2 h from ZT0 to ZT2 (Day) or ZT12 to ZT14 (Night). (*A*) Nascent proteins visualized by confocal microscopy. Box schematizes amino acid (AA) chain termination by OPP and detection with PA. Larvae treated with CHX and OPP from 3 dpf were analyzed at ZT2 as a negative control. (*B* and *C*) Quantification of OPP−PA fluorescence of somite 17 (*n* = 9 fish from three biological replicates). Signals were normalized to Control ZT0 to ZT2 samples (*B*), and compared after subtraction of CHX background (*C*). Symbols distinguish fish from different lays.

To determine how activity regulates growth, we next analyzed protein synthesis in muscle using O-propargyl-puromycin (OPP) (30). First, we examined OPP incorporation into somitic muscle and found a linear increase over 4 h (*R*^2^ = 0.89), above an unchanging background observed after pretreatment with the translational inhibitor cycloheximide (CHX) (*SI Appendix*, Fig. S4). We therefore assayed protein synthesis rate at different times of day by measuring OPP incorporation after 2 h of exposure to OPP. During the early third day (ZT0 to ZT2; when referring to time of day, we use zeitgeber time, ZT followed by hours, starting at lights on or its equivalent), OPP labeled muscle to almost twice the CHX background level (Fig. 2 *A* and *B*). Upon tricaine treatment, labeling dropped significantly by 20%, but remained well above the level in CHX (Fig. 2 *A* and *B*). We conclude that activity promotes muscle protein synthesis, but that some protein synthesis continues during the day in the absence of all muscle activity.

We next asked whether protein synthesis showed day/night variation in active fish. Strikingly, OPP incorporation was reduced in active fish at night (ZT12 to ZT14 at 3 dpf; Fig. 2 *A* and *C*). The extent of reduction in OPP incorporation between morning and evening was comparable to the effect of tricaine in the morning. To determine whether reduced activity at night or the time of day per se caused the reduction in protein synthesis, OPP incorporation was assessed in tricaine-treated larvae at night. Tricaine reduced OPP incorporation still further, such that a difference in OPP incorporation persisted between day and night in the complete absence of activity (Fig. 2 *A* and *C*). Thus, whereas physical activity promotes muscle protein synthesis during both day and night, the LD cycle promotes muscle protein synthesis preferentially during the light phase, paralleling the effects of physical activity and the LD cycle on volumetric growth.

### Diurnal Activity Acts on TORC1.

**Fig. 3.**
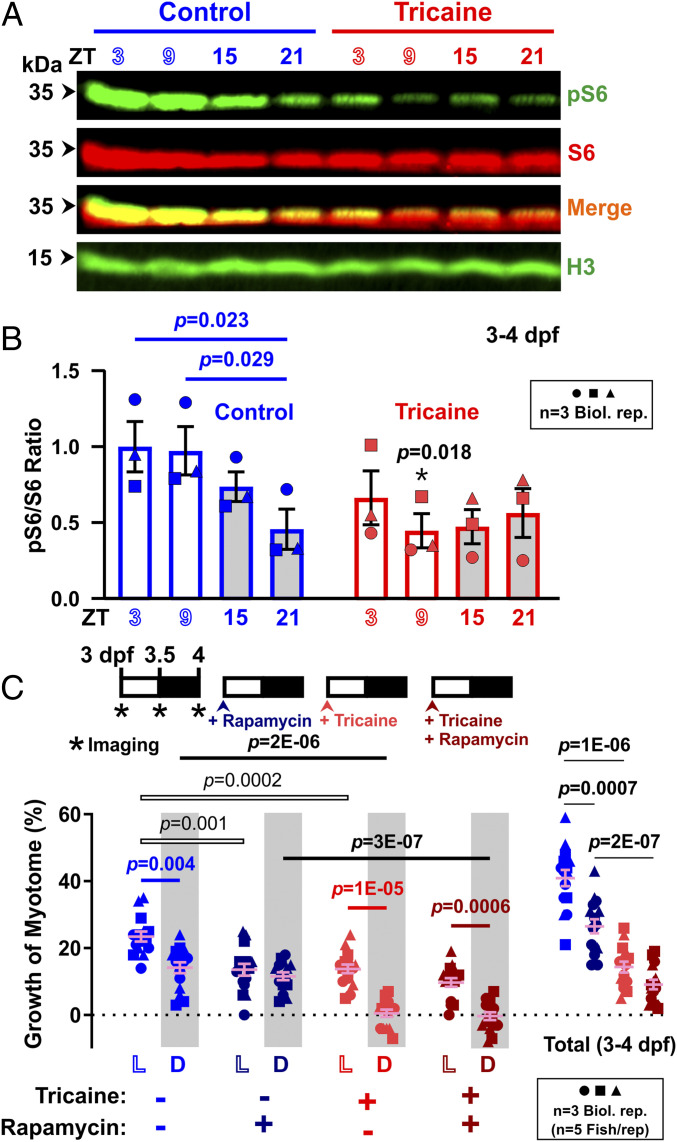
TORC1 signaling varies diurnally and is required for activity-driven daytime growth. Larvae raised under LD that were either untreated (Control, blue) or anesthetized (Tricaine, red) from 3 dpf to 4 dpf. (*A*) Western analysis of phospho-ribosomal protein S6 (pS6), total RpS6 (S6), and Histone H3 loading control (H3) in total protein extracted from whole larvae every 6 h over a 24-h period between 3 dpf and 4 dpf, at ZT3, ZT9, ZT15, and ZT21. (*B*) Quantification of pS6/S6 ratio (*n* = 3 biological replicates, shown in *SI Appendix*, Fig. S5). Ratios were normalized to control samples at ZT3. (*C*) Growth of the myotome (percent) in larvae that were also treated with either DMSO vehicle (light colors) or 10 µM rapamycin (dark colors). Symbols represent distinct lays (*n* = 15 fish, from three biological replicates). Note that DMSO data strengthen the result in Fig. 1*B*.

Muscle protein synthesis is thought to be regulated by TORC1 signaling (31). To determine whether the muscle growth observed was regulated similarly, we first examined a sensitive readout of TORC1 activity in zebrafish larvae (26), phosphorylation of ribosomal protein S6 (Fig. 3*A* and *SI Appendix*, Fig. S5). The pS6/S6 ratio showed day/night variation, being high during the day (ZT3 and ZT9) and decreasing at night (ZT15 and ZT21) between 3 and 4 dpf (Fig. 3*B*). The increased pS6 in early morning was present despite the total S6 protein content being higher at that time. As we have previously shown that a significant fraction of pS6 at earlier stages is in muscle (26), the data suggest that TORC1 activity is elevated in muscle during the day compared to the night.

The IGF1/Akt axis also drives muscle protein synthesis in some contexts (32). However, we observed no significant change in Akt phosphorylation over the diurnal cycle (*SI Appendix*, Fig. S6). If anything, slightly higher pAkt was present at night, out of phase with increased pS6 (compare Fig. 3 *A* and *B* and *SI Appendix*, Figs. S5 and S6*A*). Thus, diurnal variation in TORC1 activity appears independent of cyclic Akt activation.

We next tested the hypothesis that physical activity regulates TORC1 activity. Tricaine treatment began to reduce pS6/S6 ratio after 3 h of treatment, but had a large inhibitory effect after 9 h (Fig. 3 *A* and *B*). In contrast, tricaine had no significant effect on pS6/S6 ratio during the dark phase. This result is markedly different from the reduction in volumetric muscle growth caused by tricaine during the dark phase (Fig. 1 *B* and *C*). Nevertheless, tricaine did appear to affect ribosome content/biogenesis, as the raised level of total S6 protein present in the morning was reduced by tricaine treatment (Fig. 3*A*). These experiments suggest that TORC1 activity is higher during the day than night, and yet the diurnal rise in TORC1 activity depends on muscle contraction but not time of day.

To examine the role of TORC1 in more detail, we measured day/night muscle growth after inhibiting TORC1 with rapamycin, which we have previously shown to rapidly reduce pS6/S6 ratio in larval zebrafish (26). As before, vehicle-treated control fish grew more during the day than at night (Fig. 3*C*, blue symbols). Treatment with rapamycin reduced growth only during the day; it had no effect at night (Fig. 3*C*; compare blue and dark blue symbols). As shown previously, tricaine treatment reduced growth in both phases (Fig. 3*C*; compare blue and red symbols). Adding rapamycin to tricaine-treated fish had no significant effect on growth during either day or night (Fig. 3*C*, compare red and dark red symbols). Thus, rapamycin prevented the growth induced by activity during the day, but did not do so at night (Fig. 3*C*). We conclude that rapamycin-sensitive TORC1 activity is not required for physical activity to promote muscle growth, at least at night. During the day, however, rapamycin appears to inhibit the ability of physical activity to promote growth.

### Nocturnal Catabolism Is Suppressed by Activity.

**Fig. 4.**
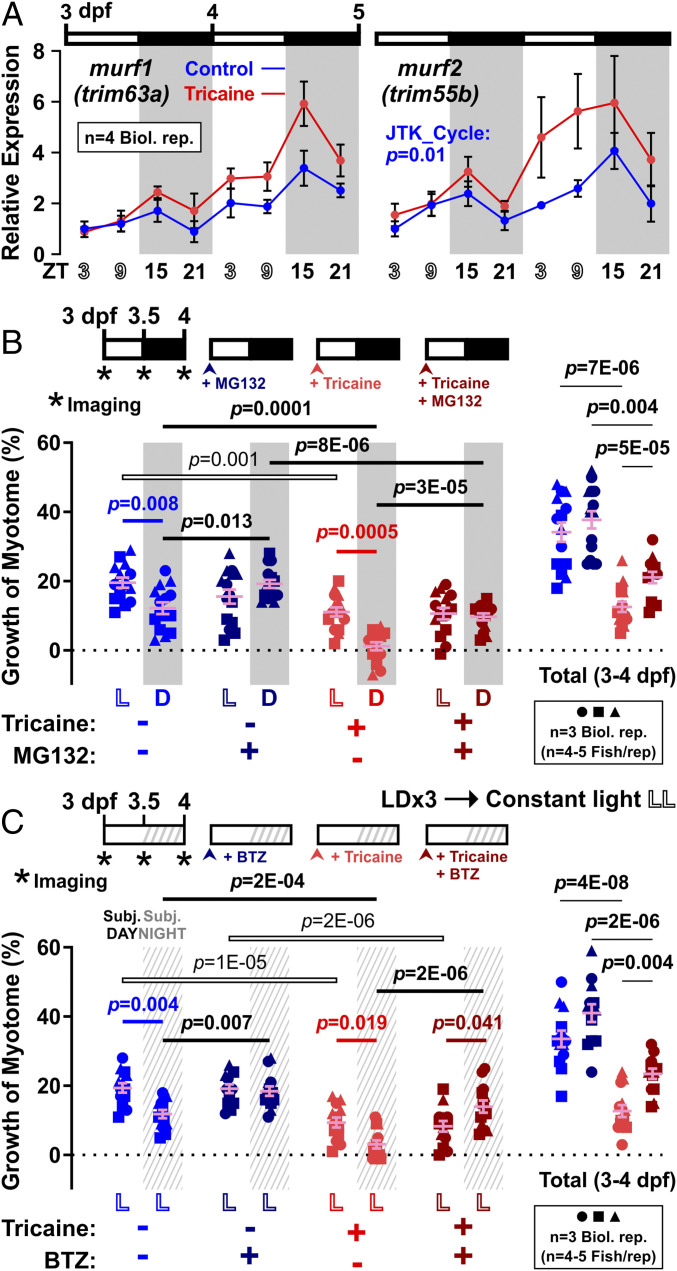
Nocturnal peak in atrogene expression parallels enhanced muscle growth at night in response to proteasome inhibitors. Larvae raised under LD were either untreated (Control, blue) or anesthetized (Tricaine, red). (*A*) Expression of atrophy-related genes *murf1/trim63a* and *murf2/trim55b* was assayed by qPCR on total RNA collected every 6 h between 3 and 5 dpf (*n* = 4 biological replicates); *ef1a* was used for normalization. Statistic represents JTK_Cycle (see *Methods*). (*B*) Growth of myotome (percent) in larvae that were also treated from 3 dpf with either DMSO vehicle (light colors) or 10 µM MG132 (dark colors). Symbols represent distinct lays (*n* = 14 or 15 fish from three biological replicates). (*C*) Growth of myotome (percent) in larvae that were entrained under LD and then at 3 dpf transferred to constant light (LD→LL) and simultaneously treated with either DMSO vehicle (light colors) or 1 µM BTZ (dark colors). Symbols represent distinct lays (*n* = 13 fish from three biological replicates). Note that DMSO data strengthen the result in Fig. 1*B*.

Several genes are known to regulate catabolism in muscle tissue by activating proteasomal proteolysis of sarcomeric proteins (33, 34). Among such genes are those encoding the Muscle RING Finger (Murf) family of Tripartite Motif (Trim) proteins that have been reported to undergo diurnal changes in mRNA level in skeletal muscle (5, 35, 36). Analysis of *murf1*/*trim63a* and *murf2/trim55b* mRNA levels in 3- to 5-dpf larvae suggested that these genes undergo day/night oscillation, such that they peak in every dark phase at ZT15 (Fig. 4*A*). The algorithm JTK_Cycle (37) confirmed at least *murf2* as a significant cycling component. Moreover, inactivity was observed to enhance the overall level of mRNA for these Murfs, but not to affect their cyclic expression. The slight increase in the baseline level of *murf* mRNAs between 3 and 5 dpf in both active and inactive fish, however, might represent a clock-independent developmental component (23). These observations raise the possibility that protein catabolism is enhanced in myotomal muscle at night.

To address the functional role of protein catabolism in muscle growth, we next used the proteasomal inhibitor MG132 to block protein degradation. We have previously shown that MG132 rapidly up-regulates known targets of the proteasome, p53 and Sqstm1, in larval zebrafish (26). MG132 alone had no significant effect on myotome growth during the day, but increased growth at night (Fig. 4*B*; compare blue and dark blue symbols). As described previously, tricaine reduced growth in both phases (Fig. 4*B*; compare blue and red symbols). Strikingly, treatment of inactive fish with MG132 still increased growth at night, and still had no detectable effect during the day (Fig. 4*B*; compare red and dark red symbols). To confirm specificity, we used a second structurally distinct proteasome inhibitor, bortezomib (BTZ), and again found an increase in growth specifically at night, independent of activity (Fig. 4*C*). These data raise the possibility that the circadian clock activates catabolism of muscle proteins at night, which counteracts on-going basal growth.

### Diurnal Variations in Muscle Growth Are Regulated by Circadian Clock.

**Fig. 5.**
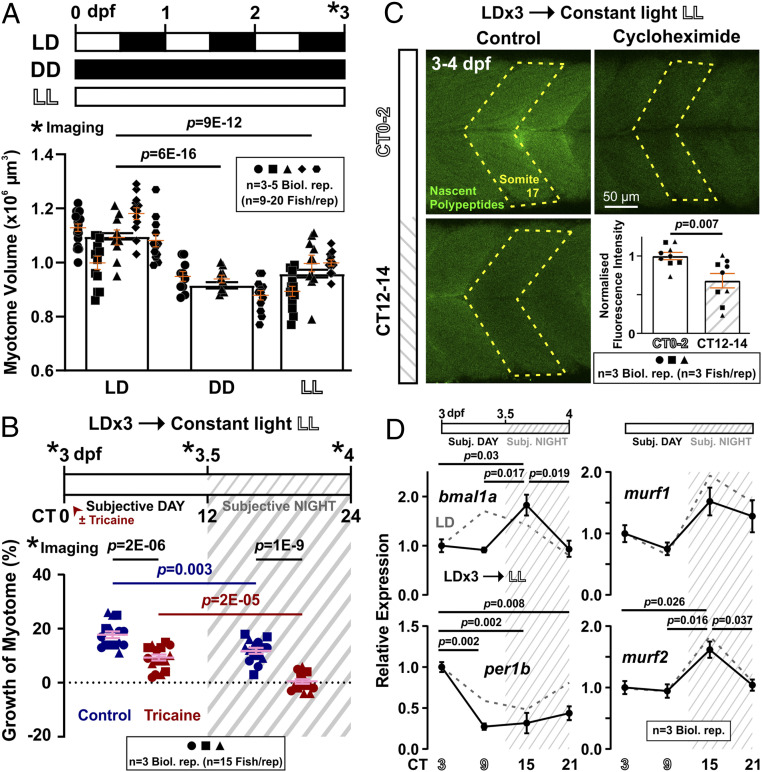
Circadian variation in muscle growth and metabolism is required for volumetric growth of myotome. (*A*) Sibling larvae collected within 1 h to 2 h of fertilization were reared under LL or DD light regimes to prevent circadian entrainment and compared to LD, as schematized. Myotome volume was measured at 3 dpf (*n* = 29 to 63 fish, from three to five biological replicates). Symbols represent distinct lays (biological replicates). Orange bars represent mean ± SEM; black boxes represent global mean. Note consistent effect despite lay-to-lay variation in absolute size. (*B*) Circadian variation in growth of myotome (percent) in larvae raised under three cycles of LD and then switched to LL between 3 and 4 dpf (LD→LL) that were either unanesthetized (Control, dark blue) or anesthetized from 3 dpf to 4 dpf (Tricaine, dark red). Symbols represent distinct lays (*n* = 15 fish from three biological replicates). (*C*) Circadian variation in nascent protein synthesis visualized at CT0 to CT2 (subjective day) and CT12 to CT14 (subjective night) between 3 and 4 dpf, in larvae that were raised under LD→LL. Graph shows quantification of OPP−PA fluorescence of somite 17 (*n* = 9 fish from three biological replicates). Signals were normalized to Control CT0 to CT2 samples after subtraction of CHX background. Symbols represent distinct lays. (*D*) Persistent circadian variation in expression of clock genes, *bmal1a* and *per1b*, and atrophy-related genes *murf1* and *murf2*, between 3 and 4 dpf in larvae raised under LD→LL (*n* = 3 biological replicates; black solid lines); *ef1a* was used for normalization. Representative traces from LD fish in separate experiments are shown as references (gray dashed lines).

Our results suggest the faster muscle growth in the day under LD is either regulated by the circadian clock or stimulated by light per se. We therefore measured muscle growth under altered light regimes. Permanent darkness (DD) or light (LL) from just after laying prevents circadian entrainment in zebrafish larvae (24), and either mutes clock function or causes clocks in each cell to run asynchronously. When larvae were exposed to either DD or LL from just after laying until 3 dpf, muscle grew about 15% less than in siblings in LD (Fig. 5*A*). Importantly, a total of 72 h of light exposure in LL yielded less muscle growth than 36 h of light exposure in LD, arguing against an acute effect of light in enhancing growth. Light is, instead, required for entrainment, as the circadian oscillation of *bmal1a*, *per1b*, and *murf* mRNAs and Bmal1 protein was perturbed under DD or LL (*SI Appendix*, Figs. S6*B* and S7*A*). Our results, therefore, indicate that a normal light-entrained circadian cycle is required for optimal myotome growth.

A critical feature of circadian organization is the perdurance of day/night variations under free-running, constant conditions (4). To test the hypothesis of true circadian control, we measured muscle growth in DD after 3 d of LD entrainment. LD→DD fish grew more in the subjective day (circadian time [CT] CT0 to CT12; when referring to time of day in the absence of zeitgeber, we use CT followed by hours, starting at subjective lights-on or its equivalent) than subjective night (CT12 to CT24), both with and without tricaine (*SI Appendix*, Fig. S8*A*), suggesting true circadian regulation. The unavoidable imaging at CT0 and CT12, however, meant total darkness was briefly interrupted; although such a stimulus is not predicted to promote less growth during the subjective night, it questions the DD free-run results. We therefore measured muscle growth in LL after LD entrainment and showed that day/night variations in growth also persisted in LL (Figs. 4*C* and 5*B*). Under constant light exposure, LD→LL larvae grew more in the subjective day but less in the subjective night, once again suggesting that light per se does not stimulate growth. As in LD fish, LD→LL fish incorporate more OPP in the subjective day (Fig. 5*C*) and express more *murf* and *atrogin1* mRNAs at night (Fig. 5*D* and *SI Appendix*, Fig. S8*B*). There is an otherwise slight phase shift of some clock genes, that is, *bmal1a* and *cry1ba* (compare LL traces with the reference LD traces), as observed previously in fish under free-running conditions (21, 38, 39), but not of *clk1*a, *per1b*, and *nr1d1* (Fig. 5*D* and *SI Appendix*, Fig. S8*B*). Thus, a higher temporal resolution might be required to determine the phase accurately. Nonetheless, all five clock genes assayed display robust oscillation under free-running conditions, suggesting sufficient entrainment of the molecular clock by three LD cycles. Together, our results indicate that day/night differences in muscle growth and metabolism are mediated by the circadian clock.

### Molecular Clock Is Required for Optimal Muscle Growth.

**Fig. 6.**
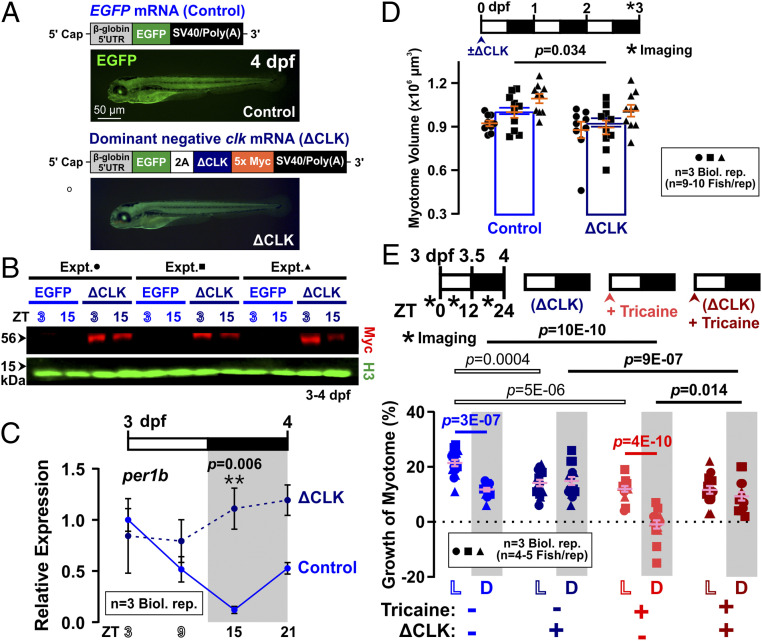
Dominant negative clock (ΔCLK) reduces daytime and enhances nighttime growth of the myotome. Control *EGFP* (light blue) and *ΔCLK* (dark blue) mRNA-injected larvae were raised under LD and analyzed between 3 and 4 dpf. (*A*) Design of *EGFP* (Control) and dominant negative *CLOCK-5xMyc* (*ΔCLK*) constructs, and their corresponding EGFP accumulation at 4 dpf after mRNA microinjection at one-cell stage. (*B*) Western analysis between 3 and 4 dpf using c-Myc antibody showing persistent presence of ΔCLK protein in *ΔCLK*-expressing larvae, but not in *EGFP* Control larvae. Histone H3 was used as loading control. (*C*) Diurnal variation in level of *per1b* mRNA prevented by ΔCLK (*n* = 3 biological replicates); *ef1a* was used for normalization. Note that Control strengthens result shown in Fig. 5*D* and *SI Appendix*, Fig. S7*B*. (*D*) Absolute myotome volume measured at 3 dpf in *α-actin:mCherryCAAX* larvae (shown in *SI Appendix*, Fig. S9*A*) is reduced by ΔCLK (*n* = 29 fish from three biological replicates). (*E*) Growth of the myotome (percent) in either unanesthetized (blue) or anesthetized from 3 dpf to 4 dpf (red) in ΔCLK (dark colors) or Control (light colors) (*n* = 14 or 15 fish from three biological replicates). Symbols represent distinct lays. Note that Control data strengthen the result in Fig. 1*B*.

To test the hypothesis of clock regulation of muscle growth further, we injected one-cell−stage embryos with mRNA encoding a dominant negative CLOCK (ΔCLK) protein or EGFP alone (control) (Fig. 6*A*). ΔCLK was previously shown to interfere with molecular clock function in zebrafish (22, 24). ΔCLK expression, as revealed by a c-Myc tag (Fig. 6*B*), disrupted the circadian oscillation of *per1b* between 3 and 4 dpf (Fig. 6*C*), and reduced muscle size of *α-actin:mCherryCAAX* fish (*SI Appendix*, Fig. S9*A*) by around 10% at 3 dpf (Fig. 6*D*), effects similar to nonentraining light regimes (Fig. 5*A* and *SI Appendix*, Fig. S7*A*). In addition, between 3 and 4 dpf, ΔCLK larvae grew less in the day compared with control larvae expressing only EGFP (Fig. 6*E*, compare blue and dark blue symbols). Hence, muscle grew similarly between day and night after clock inhibition, in both active and inactive larvae (Fig. 6*E*, dark symbols). These data suggest that the circadian clock is required for maximal daytime growth.

Active ΔCLK larvae grew somewhat more than their inactive ΔCLK siblings, at least at night (Fig. 6*E*; compare dark red and dark blue symbols). Thus, physical activity continues to promote muscle growth at night despite clock inhibition. In the absence of activity, ΔCLK increased muscle growth at night (Fig. 6*E*; compare red and dark red symbols), indicating that interfering with the clock prevents the activity-independent nocturnal atrophy. We conclude that the molecular clock is required for the circadian regulation of muscle growth, probably by increasing growth during the day and promoting atrophy at night.

### Molecular Clock Stimulates Diurnal Anabolism and Nocturnal Catabolism.

**Fig. 7.**
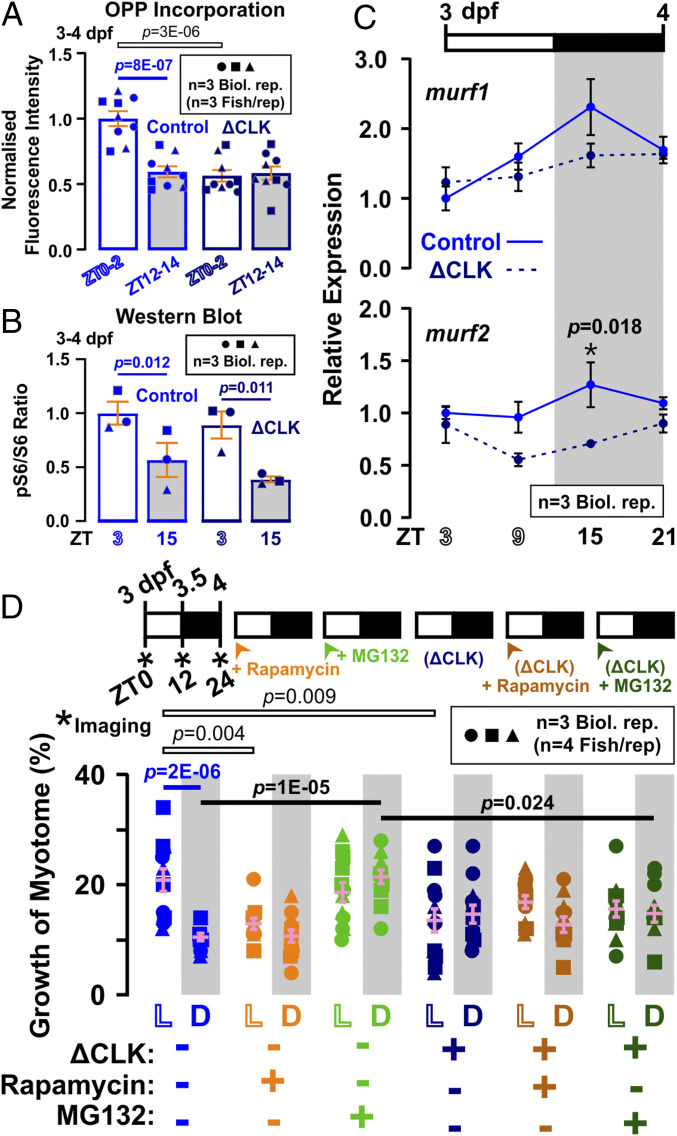
Effects of ΔCLK expression on protein metabolism in zebrafish larvae. Control (light blue) and ΔCLK (dark blue) larvae were raised under LD and analyzed between 3 and 4 dpf. (*A*) Quantification of nascent protein visualized in somite 17 by OPP incorporation at ZT0 to ZT2 and ZT12 to ZT14 (*n* = 9 fish from three biological replicates; confocal images shown in *SI Appendix*, Fig. S9*C*). Signals were normalized to Control ZT0 to ZT2 samples after subtraction of CHX background. (*B*) Quantification of pS6/S6 ratio at ZT3 and ZT15 (*n* = 3 biological replicates). Ratios were normalized to control samples at ZT3. Symbols represent distinct lays (biological replicates shown in *SI Appendix*, Fig. S9*D*). (*C*) Diurnal variation in level of atrophy-related genes *murf1* and *murf2* (*n* = 3 biological replicates); *ef1a* was used for normalization. Control strengthens results in Fig. 4*A*; ΔCLK lacks peak at ZT15. (*D*) Growth of the myotome (percent) measured between 3 and 4 dpf in *α-actin:mCherryCAAX* Control (light colors) and ΔCLK (dark colors) larvae that were treated from 3 dpf with either DMSO vehicle (blue), 10 µM rapamycin (orange), or 10 µM MG132 (green). Symbols represent distinct lays (*n* = 12 fish from three biological replicates). Note that, although the effect of ΔCLK alone to increase nighttime growth did not reach the cutoff for significance in this experimental series (*P* = 0.36) or that in Fig. 6*E* (*P* = 0.47), when both datasets were pooled, ΔCLK caused a significant increase in growth at night (*P* = 0.022). Similar pooled analysis of the reduction by ΔCLK of daytime growth also increased confidence in the difference (*P* = 2E-06).

Our findings that ΔCLK larval muscle grows less (Fig. 6) prompted study of the effects on muscle anabolism and catabolism. First, ΔCLK mRNA-injected larvae were treated with OPP, then fixed with 100% MeOH overnight to quench EGFP fluorescence that might interfere with subsequent OPP−picolyl azide (OPP−PA) detection (*SI Appendix*, Fig. S9*B*). Upon ΔCLK expression, OPP incorporation decreased significantly in the day but not at night (Fig. 7*A* and *SI Appendix*, Fig. S9*C*). Hence, disruption of the molecular clock removes the circadian difference in protein synthesis, paralleling its effects on volumetric growth (Fig. 6*E*). We show that the characteristic diurnal rise in pS6/S6 ratio remained in ΔCLK larvae (Fig. 7*B* and *SI Appendix*, Fig. S9*D*), as in permanent DD larvae (*SI Appendix*, Fig. S7*B*) and *cry1/2* double-knockout mice (40). This is consistent with our finding above that the diurnal rise in TORC1 activity depends on muscle contraction but not time of day (Fig. 3 *A* and *B*). Rapamycin, however, had no effect on muscle growth of active ΔCLK larvae (Fig. 7*D*; compare dark orange with dark blue symbols), contrary to its inhibitory effect on muscle growth in control larvae in the day (Fig. 7*D*; compare orange with blue symbols). Together, these data indicate that the extra daytime anabolism associated with growth is driven by the circadian clock and requires TORC1 activity. Moreover, the circadian clock does not act by changing TORC1 activity, but instead collaborates with TORC1-dependent physical activity to promote daytime anabolism.

In addition, ΔCLK expression attenuated the nocturnal rise in *murf* mRNAs (Fig. 7*C*). To test the hypothesis that the circadian clock also promotes nocturnal catabolism, ΔCLK larvae was treated with MG132 (Fig. 7*D*). MG132 had no effect on the growth of ΔCLK larval muscle (Fig. 7*D*; compare dark green with dark blue symbols), although it enhanced muscle growth in control larvae at night (Fig. 7*D*; compare green with blue symbols). Like MG132, ΔCLK increased the growth of larvae at night significantly when the data from Figs. 6*E* and 7*D* were pooled (*P* = 0.022, *n* = 27). We conclude that molecular clock promotes nocturnal catabolism via a ubiquitin−proteasome-dependent pathway.

## Discussion

It has previously not been possible to associate diurnal variations in muscle protein metabolism with variations in volumetric muscle growth over a single 24-h period. We report such an association in live animals in vivo. Moreover, we distinguish diurnal variation from true circadian clock-dependent growth. Diurnal variation in liver cell size and protein synthesis related to feeding has been reported, but is thought to reflect the storage function of liver, is independent of Bmal function, and has not been proved to be under circadian control (41–44). In contrast, our work provides a unified analysis leading to five major conclusions regarding circadian control of muscle growth. Firstly, we show that larval zebrafish muscle grows faster during the day than at night, and this difference reflects the balance of protein synthesis and degradation, with more synthesis during the day and more degradation at night. Secondly, these diurnal growth and metabolism differences depend on the activity of the free-running circadian clock. Thirdly, more protein synthesis during the day is driven by physical activity and the circadian clock. Fourthly, while TORC1 activity is up-regulated by physical activity and is required for clock-driven daytime growth, such growth is unlikely to arise by the clock changing TORC1 activity. Lastly, protein degradation at night is clock up-regulated, independent of both TORC1 and physical activity. To conclude, we present a testable hypothesis of how the circadian clock interacts with physical activity to control muscle growth.

### The Free-Running Circadian Clock Controls Diurnal Variation in Volumetric Muscle Growth.

In mammals, adult muscle mass maintenance is thought to be controlled by protein turnover, which displays diurnal variation. Previous studies performed in limb muscle in nocturnal animals, such as rodents, indicate that protein anabolic and catabolic activities are higher in the active phase and inactive phase, respectively (5, 6, 8, 36). Zebrafish are diurnal, more active during the day than at night (27, 28), and our data show that anabolism peaks in the day (active phase), whereas catabolism peaks at night (inactive phase). Importantly, we show that these metabolic oscillations 1) occur in the absence of physical activity and 2) are absent in ΔCLK fish, as in loss-of-function clock mutant mice (6, 18, 36). Although these findings suggest a role of the circadian clock, no previous study has been able to demonstrate the functional significance of the circadian clock per se for volumetric muscle growth. To distinguish true circadian clock control from the effects of other diurnal variation (such as lighting, physical activity, or feeding), it is essential to show three things: 1) continued oscillation under free-running constant conditions, 2) dependence on the core clock molecular mechanism, and 3) dependence on entrainment of the core clock. By demonstrating continued diurnal growth variation under the constant conditions of LD→LL or LD→DD, the inhibition of growth variation by ΔCLK and diminished growth when the clock is not entrained (under permanent dark or light conditions), and reversal of growth phases when the light cycle is reversed, our study fulfills the criteria for demonstration of true circadian clock-dependent muscle growth. Moreover, as ΔCLK abolishes the ability of MG132 to enhance muscle growth specifically during the night, our data indicate that at least some circadian clock-dependent metabolic oscillations contribute to volumetric muscle growth.

We have not directly addressed the cellular mechanism of volumetric muscle growth in the current report. However, we have previously shown that most muscle growth in somites 16 to 20 between 1 and 6 dpf is caused by fiber hypertrophy, increase in cross-sectional area, rather than new fiber formation (20). The theoretical consideration that, at their inception, new fibers are smaller than preexisting fibers also indicates that new fiber formation is unlikely to contribute much to volumetric growth over a 24-h period. Thus, we conclude that the circadian variation in muscle growth likely arises from differences in the rate of hypertrophy of existing fibers.

In many circadian studies, it is difficult to eliminate rhythmic feeding as a potential source of circadian variation in muscle metabolism and growth (7, 11). We eliminate such feeding cues in two ways. Firstly, larval zebrafish do not feed until 5 dpf (45) Secondly, no food was provided for larvae in our study. Whether nutrient availability within larvae shows diurnal variation is unknown. At the stages examined, nutrition and substrates for muscle anabolism were shown to derive from the yolk constantly via the circulation (46), but we found no evidence of diurnal variation in yolk consumption.

Our study was largely focused on the fourth day of development, a stage after hatching and also one at which general anesthetic can be administered for 24 h without significant death or major impairment of later life. By reversing light cycles, we show that developmental stage differences are not responsible for the circadian muscle growth difference observed. We further eliminated temperature and gender as requirements for circadian difference in muscle growth, as larvae are gender neutral and were reared under a constant temperature environment throughout the experiments.

In line with our observations that altered light regimes and clock inhibition reduce larval muscle growth, a previous study showed that altered light regimes decrease body length of zebrafish larvae (47). In adult, moreover, clock and Murf genes cycle in a similar way to what we observe in larvae (35). Furthermore, artificial selection of wild-type zebrafish for lines with large and small muscle mass selected for distinct expression or polymorphisms in *per* and *cry* genes, key components of the circadian clock (48). Taking these findings together, the circadian clock appears to be important for muscle growth and homeostasis throughout larval and adult life. Whether the clock functions intrinsically within muscle cells or operates elsewhere in the body to entrain muscle must await muscle-specific clock disruption in fish.

### Physical Activity Drives Muscle Anabolism and Growth during the Day in Cooperation with the Circadian Clock.

Animals display a circadian pattern of activity, and activity per se stimulates muscle growth (26). Hitherto, it has not been possible to remove muscle contractile activity as a confounding factor during the study of circadian regulation of muscle metabolism and growth (36). We show that, in fish, inhibition of all muscle activity decreased anabolism and hence growth in both day and night to a similar extent. Nevertheless, inactive muscle maintains a circadian protein turnover and growth difference. This argues that the circadian clock directly controls muscle growth independent of any effect on physical activity or muscle use.

**Fig. 8.**
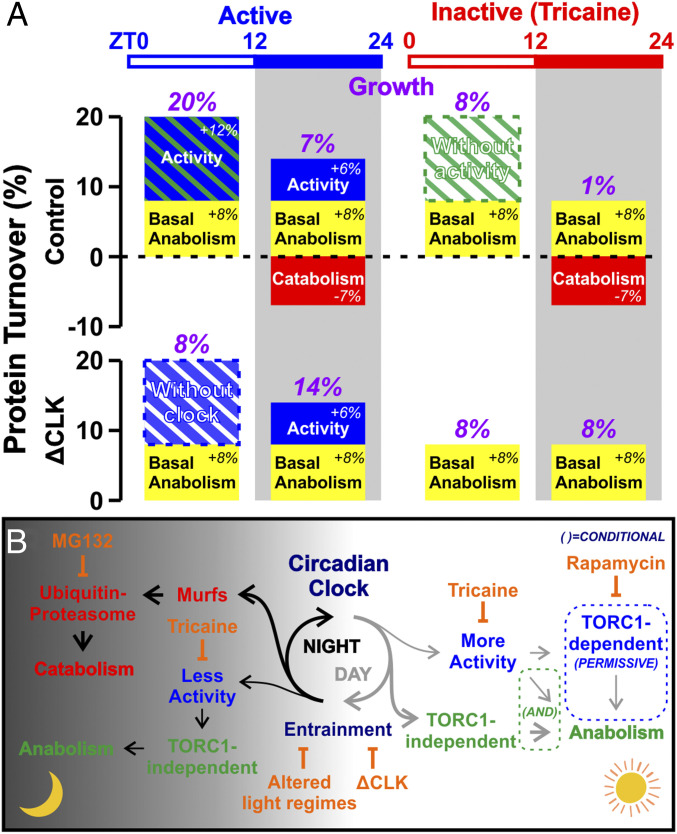
Models of how balance of anabolism and catabolism yields observed muscle growth through interaction of circadian and physical activity-dependent regulation. (*A*) Circadian muscle growth model. The *y* axis represents net protein turnover of cellular material, that is, hypertrophy (positive) or atrophy (negative). The *x* axis represents condition. Observed (and predicted net, from summed colored bars) growth over 12-h period is shown above (numbers in purple). Blue represents anabolism driven by activity. Green represents circadian clock-driven anabolism that is increased during the day. Striped blue and/or green represent the cooperative requirement of the two components, activity and clock, to drive extra daytime growth. Red represents catabolism induced by the circadian clock at night. Yellow represents activity- and clock-independent basal intrinsic anabolism/growth. During a normal LD cycle (*Upper*), in the presence of activity, muscle growth is fastest in the day, as physical activity cooperates with circadian clock to drive extra daytime anabolism. At night, anabolism reduces and catabolism increases, leading to slower growth, but activity still promotes some growth. In the absence of activity, muscle growth is still faster in the day than at night, although anabolism is reduced. This is because clock still drives nocturnal catabolism, but is insufficient to drive extra daytime anabolism for maximal growth (see striped green surrounded by dashed line). In the absence of a functional clock (*Lower*), muscle growth in the day is reduced compared with active control, because physical activity alone is insufficient to drive extra anabolism for maximal growth (see striped blue surrounded by dashed line). Growth at night, however, increases as nocturnal catabolism is eliminated. In the absence of activity, muscle growth in the day is unaltered when compared with inactive control. At night, growth again increases, due to the elimination of nocturnal catabolism. Hence, growth is similar between day and night in inactive ΔCLK muscle. Overall, optimal growth and protein turnover is only achieved in active muscle with a functional clock. (*B*) Proposed mechanisms of clock regulation of metabolism. Gray arrows, daytime pathways; black arrows, nighttime pathways. Note that TORC1 activity is not absolutely required for anabolism/growth; TORC1 activity, however, permits extra anabolism and hence faster growth in the day (blue dashed box), but only when both physical activity and clock are present (green dashed box).

The way in which physical activity promotes growth is, however, different during the day from its action at night. As shown in Fig. 6*E*, blockade of either the circadian clock or physical activity alone causes a large and indistinguishable reduction in daytime growth of around 50%. It should be noted that ΔCLK caused no obvious change in larval motility. These effects are not additive, implying that they affect the same process. No such cooperation was observed at night (see below). Thus, during the day, the clock and physical activity appear to act as a logical AND gate; both are required for the extra daytime growth above a basal level (Fig. 8).

We examined the mechanism by which the clock and physical activity promote muscle growth. Blockade of either stimulus reduces protein synthesis, as measured by short-term OPP incorporation into muscle tissue, by about 50%. As muscle still grows by about 50% of the normal amount in the presence of dual blockade (of both clock and activity), it seems there is a basal level of daytime muscle growth in zebrafish larvae that we have been unable to prevent by any manipulation, which parallels the 50% of protein synthesis that remains. We note that this basal rate of anabolism may simply reflect evolutionary selection for rapid growth of developing larvae. These data indicate that both physical activity and the circadian clock cooperate to promote muscle anabolism, at least at the level of protein synthesis.

### TORC1 Activity Is Required for Muscle Growth during Day but Not at Night.

TORC1 activity has been implicated in muscle anabolic growth in a wide variety of situations (31). Strikingly, by using the inhibitor rapamycin, we obtained evidence that TORC1 activity is essential for about half of the normal muscle growth during the day, but is not required at night. We therefore investigated the effect of physical activity and the circadian clock on TORC1 activity by analyzing pS6/S6 ratio, as a simple and sensitive initial readout (26). Whereas blocking physical activity reduced pS6/S6 ratio, blocking the circadian clock caused no detectable change, even though TORC1 inhibition has been shown to lengthen clock period or decrease amplitude in some circumstances (49). Given that the stimuli cooperate to promote muscle growth, this result suggests that such cooperation occurs downstream of any effect of activity on TORC1. Indeed, our findings are consistent with the view that TORC1 activity is not a prime controller of daytime muscle growth, but is either a permissive signal acting in addition to the main cooperative pathway or a gain control on such a pathway (Fig. 8*B*).

Growth appeared to be rapamycin insensitive in inactive fish, suggesting that the activity-independent anabolism and growth, both day and night, are TORC1 independent. The total content of ribosomal protein S6 in larvae varied across the diurnal cycle, being higher during the day, and this diurnal difference was variably diminished by inhibition of physical activity. Thus, reduction of pS6/S6 ratio in inactive muscle may reflect both a reduction in absolute level of S6 and TORC1 kinase activity acting through the p70S6 kinase pathway. It is notable that Bmal1, a major clock component that peaks in the inactive phase, can promote both ribosomal RNA biogenesis and overall protein synthesis (50, 51). Moreover, at night, blockade of physical activity had no effect on pS6/S6 ratios, yet diminished growth, again indicating that the growth promoting effect of physical activity does not act via TORC1. Indeed, at night, rapamycin had no effect on muscle growth. Further understanding of the role of TORC1 in muscle growth in our system will require the use of more specific inhibitors, genetic manipulation, and consideration of effects on the various TORC1 target pathways, including autophagy (52).

### The Circadian Clock Diminishes Growth at Night by Promoting Catabolism.

Overall growth is lower at night because the circadian clock enhances proteolysis in this phase. Enhanced muscle proteolysis in the inactive phase has been observed in mammals (5–7, 11), and, in *Drosophila*, proteolysis is associated with improved muscle function and increased longevity (53, 54). Remarkably, treatment of larvae with the proteasomal inhibitors MG132 or BTZ increases muscle growth at night but has no discernible effect during the day. Moreover, this nocturnal effect is not blocked by making muscle entirely inactive, but is lost in ΔCLK-injected larvae, which strongly suggests that the circadian clock promotes proteasome-dependent catabolic events at night that counteract anabolic growth signals.

Mechanistically, ΔCLK abolishes the peak of mRNA accumulation from two zebrafish Murf genes, *trim63a* and *trim55b*, which normally occurs early in the night phase. These ubiquitin E3 ligases, Murf1 and Murf2, regulate muscle atrophy in other species and undergo cyclic gene expression, accumulating in the inactive phase in a variety of species (5, 8, 35, 36), just as we show in zebrafish larvae. Additionally, we show that these genes and *fbxo32* are under true circadian control. Both Murf genes are most highly expressed in muscle of adult fish, Murf1 is localized to sarcomeres, and the Murfs are thought to mediate muscle protein turnover during atrophy (55, 56). We therefore hypothesize that the circadian degradation of proteins in muscle not only maintains muscle quality long term but also helps to regulate muscle fiber size. Whether other important proteostatic regulators, such as autophagy, are also under circadian control remains to be determined.

As mentioned above, physical activity enhances both growth and protein synthesis in fish muscle at night, although it has no significant effect on pS6/S6 ratio. In contrast to the situation in daytime, however, exercise-induced growth does not require cooperation with the circadian clock; the effect of each regulator is additive. Our findings do not address the important issue of the quantitative and qualitative aspects of exercise. Larval zebrafish sleep at night, and swim less (57). Our measurements of size changes and protein synthesis are not currently precise enough to determine whether the growth-promoting effect of activity 1) depends on the type or quantity of activity or 2) differs between day and night. However, the data contain a trend that leads us to hypothesize that the positive effect of activity is greater during the day, when activity cooperates with positive clock-derived signals to promote growth (Fig. 8*A*).

### Model for Circadian Muscle Growth and Mechanism of Circadian Regulation of Metabolism.

In conclusion, our findings significantly advance understanding of the interaction between the circadian clock, physical activity, and the role of feeding on skeletal muscle growth, at least in our model system. In both fish and mammals, various clock and Murf genes show diurnal cycles in adult muscle (5, 8, 35, 36). It remains to be seen whether the principles discerned in the current work apply more broadly to adult zebrafish muscle and to other species. If they do, deeper study may increase understanding of the effects on muscle growth and maintenance of aging, in which the circadian clock weakens and exercise diminishes (18, 58, 59). We present a hypothetical quantitative interpretation of how the circadian clock, exercise, and the balance of anabolism and catabolism contribute to the growth of larval zebrafish muscle (Fig. 8*A*). In terms of molecular mechanism, we have made some progress (Fig. 8*B*), but much remains to be discerned. In particular, whether the circadian clock operates autonomously within zebrafish muscle or depends on cues from elsewhere in the body and how the clock and physical activity signals are integrated into the control of anabolic and catabolic metabolism need further investigation.

## Methods

### Zebrafish Lines and Maintenance.

Zebrafish were reared at 28.5 °C on a 14-h (09:00 to 23:00)/10-h light/dark cycle, with staging and husbandry as described (60). Newly fertilized embryos were obtained by pairwise natural spawning, cleaned, and transferred within 1 h to 2 h to incubators at 28.5 °C fitted with timed LED lighting systems on a 12-h light (L; 09:00 to 21:00):12-h dark (D; 21:00 to 09:00) cycle, or other regimes as described in text and *SI Appendix*, *Supplementary Methods*. No more than 80 fish were present in each dish, and chorion debris and dead or sick fish were removed daily where the light regime permitted. All experiments were performed in accordance with licenses held under the UK Animals (Scientific Procedures) Act 1986 and later modifications and conforming to all relevant guidelines and regulations.

### Embryo Manipulation.

*Tg(Ola.Actb:Hsa.HRAS-EGFP)*^*vu119*^ (61) and *Tg(acta1:mCherryCAAX)* (62) were maintained by backcrossing on the AB wild-type background. Tricaine (Sigma-Aldrich) was prepared as described (60). Drugs were prepared in dimethyl sulfoxide (DMSO) at stock concentrations of 5 mM (rapamycin; Enzo Life Sciences) and 1 mM (MG132; AlfaAesar and BTZ; APExBIO), stored at −20 °C and diluted to 10 µM (rapamycin and MG132) or 1 µM (BTZ) in either fish water alone or tricaine-containing fish water when used. Control groups were treated with 0.2 to 1% DMSO alone.

### OPP Protein Synthesis Assay.

Experiments were performed using Click-iT Plus OPP Protein Synthesis Assay Kit with Alexa Fluor 488 PA (Invitrogen) (*SI Appendix*, *Supplementary Methods*). Quantification of the mean fluorescent intensity was performed on single optical sections at equivalent depth in whole somite 17 using Fiji (NIH). Normalized signals were calculated by subtracting mean signals measured in negative control.

### Imaging.

Bright-field and wide-field fluorescence imaging was performed under a Leica MZ16F with Imaging Development Systems Gmbh iDS camera. For confocal imaging, larvae were mounted in 0.8 to 1% low melting point agarose, and data were collected on the somites 17 and 18 near the anal vent on a Zeiss LSM 5 Exciter microscope equipped with 20×/1.0W objective and subsequently processed using either Volocity 6.3 (Perkin-Elmer), Fiji (NIH), or ZEN (Zeiss) software. For live imaging, mounted larvae were transiently anesthetized with tricaine. Immediately after the scan, larvae were washed and then separately housed in 24-well or 96-well plates to track individual myotome growth. Medium in each well was changed every 12 h. Myotome volume was calculated as described (19, 20) and schematized in *SI Appendix*, Figs. S1*A* and S9*A*.

### Protein Detection.

Protein extraction, sodium dodecyl sulfate polyacrylamide gel electrophoresis, and Western blotting were performed as described in ref. 63 and in *SI Appendix*, *Supplementary Methods*. Primary antibodies used were Akt (pan) (1:1,000; #2920, Cell Signaling), phospho-Akt^Ser473^ (1:1,000; #4060, Cell Signaling), S6 ribosomal protein (1:1,000; #2317, Cell Signaling), phospho-S6^Ser240/244^ ribosomal protein (1:1,000; #5364, Cell Signaling), Bmal1 (1:1,000; #ab93806, Abcam), c-Myc (1:1,000; #MCA2200GA, Bio-Rad), actin (1:1,000; #A2066, Sigma-Aldrich), and histone H3 protein (1:1,000; #ab1791, Abcam). Secondary antibodies used were Alexa Fluor 488 GoatαMouse IgG(H+L) (1:1,000; #A-28175, Invitrogen), Alexa Fluor 633 GoatαRabbit IgG(H+L) (1:1,000; #A-21050, Invitrogen), horseradish peroxidase (HRP) GoatαMouse IgG(H+L) (1:5,000; #AP308P, Sigma-Aldrich), and HRP GoatαRabbit IgG(H+L) (1:5,000; #AP307P, Sigma-Aldrich). The pS6/S6 and pAKt/Akt ratios were normalized to control samples at ZT/CT3.

### RNA Detection.

RNA extraction, RT-PCR, and qPCR were performed and results analyzed as described in ref. 63 and in *SI Appendix*, *Supplementary Methods*. Primers used are listed in *SI Appendix*, Table S1.

### Microinjection.

Around 3 nL of *EGFP* or *EGFP-2A-ΔCLK-5xMyc* mRNA at 200 µg/µL to 300 µg/µL were microinjected into embryos at the one-cell stage in equal molar amount (i.e., ∼1.3 pmol).

### Statistical Analyses.

Quantitative analysis on images was performed using Fiji (NIH). Statistical analysis was performed on Prism 8.2.1 (GraphPad). See *SI Appendix*, *Supplementary Methods* for details of tests performed.

## Supplementary Material

Supplementary File

## Data Availability

All data supporting the findings of this study are available within the article and its *SI Appendix*.
