# Supplementary material for: Circadian regulation of muscle growth independent of locomotor activity
Source: Proc Natl Acad Sci U S A. 2020 Nov 23;117(49):31208–18. doi: 10.1073/pnas.2012450117 (PMC7733834; doi:10.1073/pnas.2012450117)
Supplement: Supplementary File [file pnas.2012450117.sapp.pdf]

PNAS
www.pnas.org

Supplementary Information for

Circadian regulation of muscle growth independent of locomotor activity.

Jeffrey J. Kelu, Tapan G. Pipalia and Simon M. Hughes[1]*

[1]Randall Centre for Cell and Molecular Biophysics, King's College London, SE1 1UL, UK.

* Author for correspondence s.hughes@kcl.ac.uk

Randall Centre for Cell & Molecular Biophysics, New Hunt's House, Guy's Campus, King's College London, SE1 1UL, UK

This PDF file includes:

- Supplementary Methods
- Figures S1 to S9
- Table S1
- Supplementary Information References

www.pnas.org/cgi/doi/10.1073/pnas.2012450117

Supplementary Methods

Light and temperature control

The intensity of light was maintained at around 100 μW/cm^2 (1). A temperature change of 2ºC or more can entrain the circadian clock under otherwise constant conditions (2, 3). Incubator temperature was monitored with a thermocouple probe every 10 s for 24 h for an entire light/dark cycle, revealing a maximum temperature variation of 0.37ºC. As the mean temperature in the light/day period was <0.1ºC higher than that in the dark/night, any subtle temperature changes are insufficient to entrain the clock under constant light or dark conditions.

O-propargyl-puromycin (OPP) assay

To assess the incorporation rate of OPP, larvae at 3 dpf were treated with 50 μM Click-iT® OPP Reagent for 1, 2, 3, or 4 h before fixing with 2% paraformaldehyde (PFA) at room temperature for 30 min. As a negative control, larvae were treated with 200 μg/mL cycloheximide (CHX; Sigma-Aldrich) for 1 h prior to addition of OPP. To assess diurnal OPP incorporation, larvae were treated with Click-iT® OPP Reagent for 2 h at either 3 dpf (ZT/CT0-2) or 3.5 dpf (ZT/CT12-14), before fixing with either PFA or 100% MeOH (at -20ºC overnight). Larvae were also treated with CHX for 1 h prior to labelling as a negative control. Fixed larvae were bleached in 10% H_2O_2 and 5% formamide solution to remove pigment as described (4), and then permeabilised with phosphate-buffered saline (PBS) containing 0.1% Triton X-100 and 1% DMSO. Permeabilised larvae were then labelled with Click-iT® Plus OPP reaction cocktail (Invitrogen) for 1-2 h in dark in room temperature according to manufacturer's instructions. In some experiments, Hoechst 33342 (1:10000; H3570; Invitrogen) was used as a counterstain. Labelled larvae were then washed thoroughly with PBS, mounted and subjected to confocal imaging with no saturated or black pixels. Within an experiment, all fish were imaged on identical settings.

Protein extraction, SDS-PAGE, and Western blotting

Total protein was extracted from pools of twenty larvae via sonication in solution mixtures containing Tissue Extraction Reagent I (Invitrogen), cOmplete™, EDTA-free Protease Inhibitor Cocktail Tablets (Sigma-Aldrich), and phenylmethylsulfonyl fluoride (PMSF). Protein extracts were then pelleted by centrifugation, after which the supernatant was mixed with Laemmli Sample Buffer (Bio-rad) and 2-mercaptoethanol (Bio-rad) and heated at 95ºC for 5-10 min, before subjecting to SDS-PAGE analysis. Protein extract equivalent to 1-2 larvae was loaded per lane. SDS-PAGE was performed using either handcast polyacrylamide gels or Mini-PROTEAN® TGX Precast Gels (Bio-rad), and separated proteins were then transferred to nitrocellulose membranes, blocked in either 5% non-fat dry milk or 5% BSA in Tris-buffered saline (TBST), incubated in primary and secondary antibodies at 4ºC overnight and at room temperature for 1-2 h, respectively. Amersham ECL Prime Western blotting detection reagent was used for HRP detection (GE Healthcare). All signal detection was performed using ChemiDoc™ Imaging System (Bio-rad) and analysed on Image Lab™ Software (Bio-rad).

RNA extraction, RT-PCR and qPCR

Total RNA was extracted from pools of five larvae using Tri-reagent (Sigma-Aldrich) via sonication, isolated via phase separation using 1-bromo-3-chloropropane, precipitated using 2-propanol and washed using 75% ethanol. Extracted RNA was treated with TURBO DNase (Invitrogen) for 15-30 min at 37ºC. Purified total

RNA (500 ng) was then reverse transcribed using Eurogentec Reverse Transcriptase Core Kit (Eurogentec) with both random and oligo-dT primers following manufacturer's instructions. For qPCR, technical triplicates were performed on 5 ng of cDNA using Takyon Low ROX SYBR 2X MasterMix blue dTTP (Eurogentec) on a ViiA™7 thermal cycler (Applied Biosystems). ΔCt was calculated by subtracting the Ct value of the *ef1a* housekeeping gene from that of the target gene. ΔΔCt of each target gene was then calculated by subtracting the average ΔCt of samples, and relative gene expression calculated as $2^{-\Delta\Delta Ct}$ (5), followed by normalising to control samples at ZT3.

Molecular cloning and *in vitro* transcription

PCR was performed to clone *EGFP-2A-ΔCLK-5xMyc* fragment from *pT2-aanat2-EGPF-2A-ΔCLK-5xMyc* (6) using Q5 Hot Start High-Fidelity DNA Polymerase (NEB). To generate *pCS-EGFP-2A-ΔCLK-5xMyc*, the *EGFP-2A-ΔCLK-5xMyc* fragment was subcloned into a *pCS* backbone (from *pCS-mCherryCAAX*) using NEBuilder HiFi DNA Assembly Master Mix (NEB) according to manufacturer's instruction. Sequence integrity was then confirmed by Sanger sequencing (Genewiz). Primers used are listed in Table S1. Capped mRNAs were synthesised using mMESSAGE mMACHINE™ SP6 Transcription Kit (Invitrogen) using NotI-HF (NEB)-linearised *pCS2+-EGFP* and *pCS-EGFP-2A-ΔCLK-5xMyc* as templates. Synthesised mRNAs were subsequently purified with phenol/chloroform and then resuspended in nuclease-free water.

Statistical tests

Tests for normality were performed using D'Agostino and Pearson test (omnibus K2), taking *p*-values >0.05 to indicate that the distribution of measured myotome volumes was not inconsistent with a Gaussian (normal) distribution. We, therefore, presume that all further measurements are normally distributed, justifying use of two-tailed paired sample (experiment/day) unbalanced (due to slight variation in number of embryos analysed in each condition or experiment) ANOVA with two factors ('experiment/day' and 'treatment'), followed by Bonferroni's post-hoc tests. All data were shown with bars representing mean ± standard error of the mean (SEM). All experiments were performed on at least three independent biological replicates. *p*-values for rejection of the null hypothesis of no difference between groups are indicated above lines ending at the columns compared (for example, 1E-06 abbreviates 1×10^{-6}). For the detection of rhythmic components in qPCR data sets, JTK_Cycle, a non-parametric algorithm (7) was applied, using the statistical computing software R version 3.5.2.

Figure S1

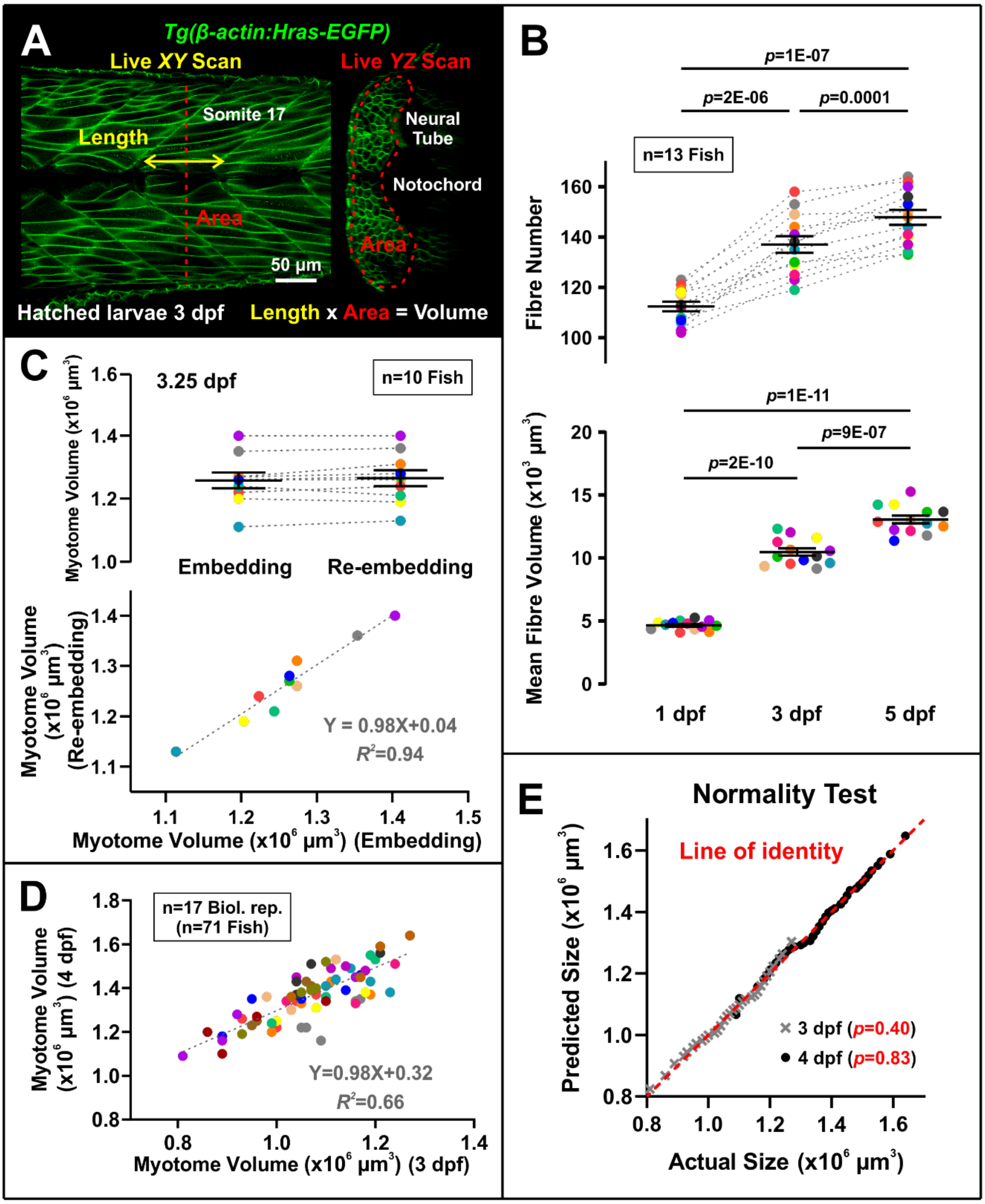

Fig. S1. Highly-reproducible live assay shows myotome grows by increase in both muscle fibre volume and number. A. *β-actin:Hras-EGFP* transgenic larvae were transiently anaesthetised, mounted, and scanned from left lateral view (dorsal to top) in *XY* and *YZ* optical confocal sections of somite 17 to measure myotome dimensions and calculate volume. **B.** Embryos/larvae were repeatedly imaged to assess variation in growth rate between individuals. Increase from 1-5 dpf in both fast fibre number (~27%) and mean fast fibre volume (~280%, calculated as [myotome volume]/[fibre number]) in somite 17 of individual fish (indicated by colour, corresponding to the myotome volume data shown in Fig. 1A) (n=13 fish). **C.** Repeated re-embedding and re-scanning of single larvae within 1 h (individuals indicated by colour) showing high concordance (<3% variation, R^2=0.94) in measured somite 17 myotome volume (n=10 fish). Note the slight trend towards increase

in mean volume, consistent with growth between scans. Black bars represent mean ± SEM. **D.** Somite 17 myotome volume of individual larvae at 3 dpf was compared with the same individuals at 4 dpf (n=71 fish). Colours show individuals from 17 biological replicate lays. **E.** D'Agostino and Pearson Normality Test shows the lack of deviation of data in Fig. S1D from a Gaussian (normal) distribution. *p*-values >0.05 accept the null hypothesis that all values were sampled from a population that follows a Gaussian distribution. Symbols distinguish myotome volume measure at 3 dpf and 4 dpf.

Figure S2

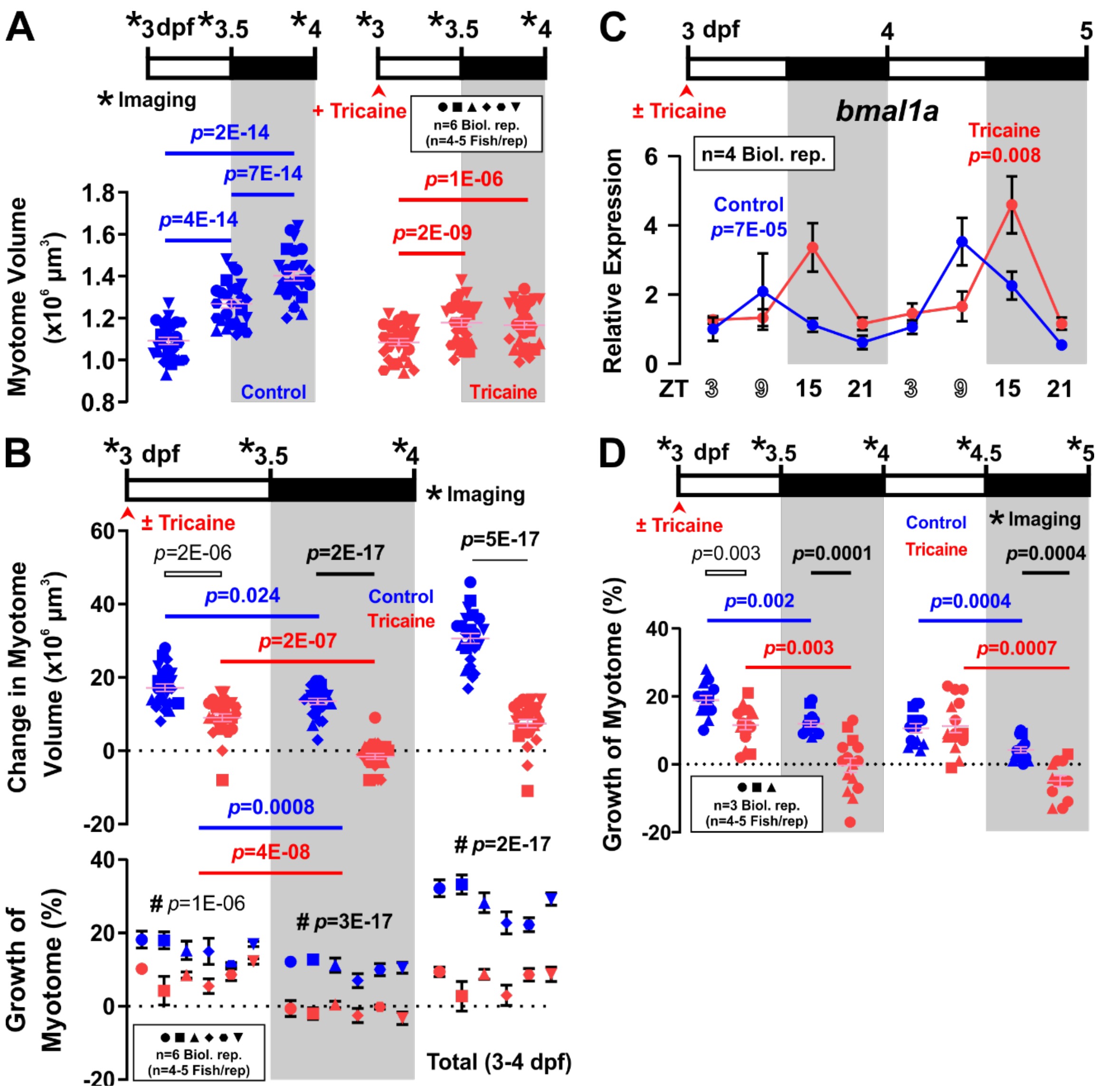

Fig. S2. Circadian growth difference in absolute myotome volume across lays and developmental stages. A,B. Data underlying that presented in Fig. 1B as myotome growth (%). Larvae raised under LD (n=25-26 fish from 6 biological replicates) were either untreated (Control, blue) or anaesthetised (Tricaine, red) from 3-4 dpf. Confocal imaging was performed every 12 h (*) over the 24 h period to measure somite 17 myotome volume of individual fish at each timepoint (A), absolute change in myotome volume for each fish across all six lays (B upper) and mean ± SEM for each individual lay (B lower). Note the consistency in growth and effects of time and anaesthetic, despite differences in absolute mean size between lays. # indicates significant differences between control and anaesthetised fish. **C.** Expression of core clock gene *bmal1a* was assayed by qPCR every 6 h between 3 and 5 dpf from untreated (Control, blue) and anaesthetised (Tricaine, red) larvae raised under LD (n=4 biological replicates). *ef1a* was used for normalisation. Statistic represents JTK_Cycle (see Methods). **D.** Growth of myotome (%) measured between 3 and 5 dpf in untreated (Control, blue) and anaesthetised (Tricaine, red) larvae raised under LD (n=12-15 fish from 3 biological replicates). Note the declining growth rate with age, consistent effect of anaesthetic and persistent circadian difference with or without anaesthetic. Symbols distinguish biological replicates from separate lays.

Figure S3

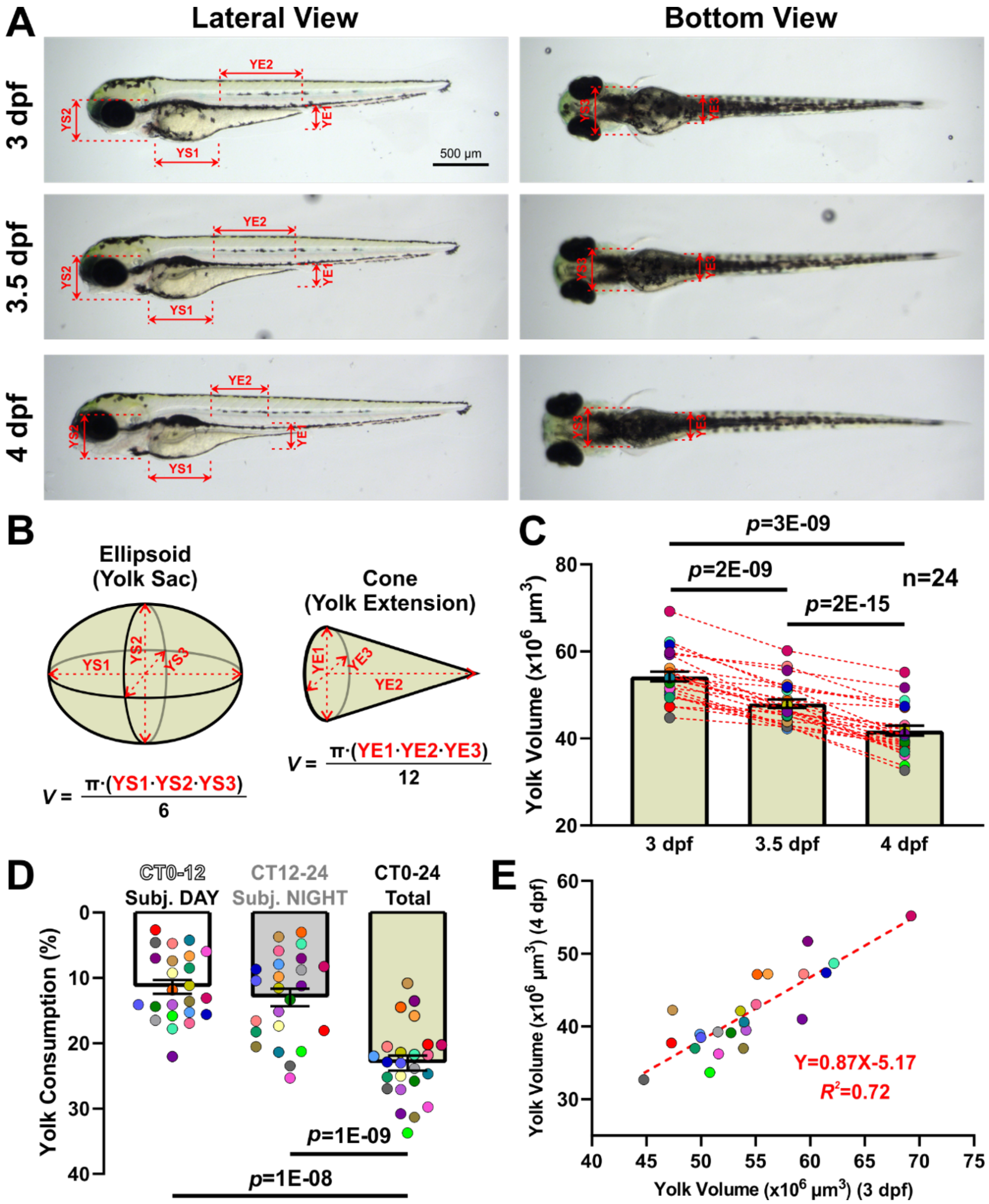

Fig. S3. Yolk consumption is indistinguishable between day and night in live zebrafish larvae. Zebrafish larvae were raised under 3 cycles of LD and then transferred to constant light (LD→LL) at 3 dpf, shown in left lateral view (dorsal to top) and bottom view (anterior to left). **A.** Single unmanipulated fish were transient anaesthetised, mounted, and imaged repeatedly every 12 h between 3 and 4 dpf to assess the change in yolk size over time. Several dimensions of the yolk sac and yolk extension were measured (see corresponding red lines). **B.** Formulae used to calculate the total volume of the yolk based on the assumed shape of an ellipsoid yolk sac and conical yolk extension. **C,D.** Absolute yolk size (C) and percentage change (D) from 3 to 4 dpf (n=24 fish). **E.** Yolk volume of individual larvae at 3 dpf was compared with the same individuals at 4 dpf. Colours show different individuals. Black bars represent mean ± SEM.

Figure S4

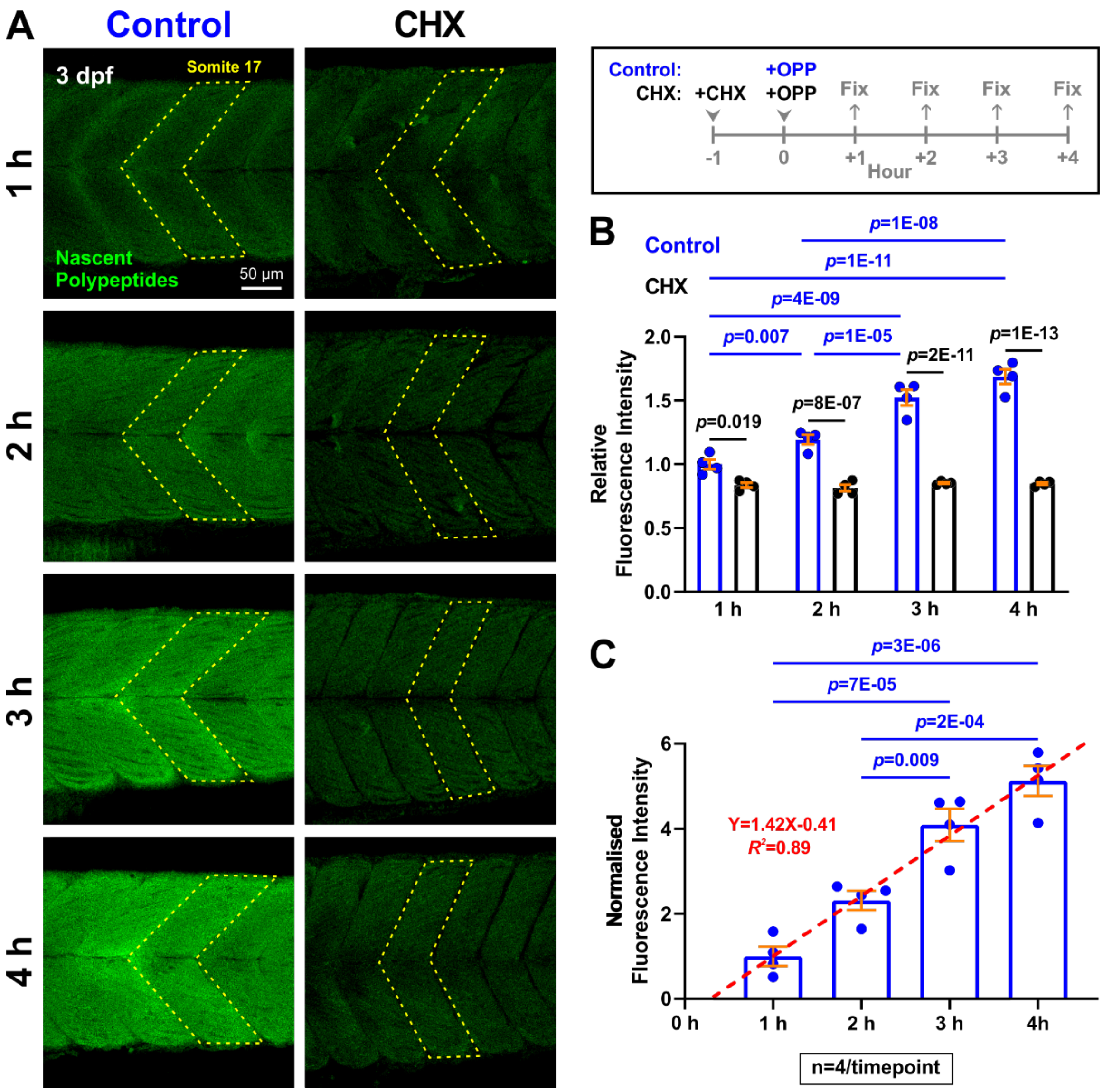

Fig. S4. Steady incorporation of OPP into zebrafish myotome *in vivo* depends on protein synthesis. Larvae at 3 dpf were treated with OPP for 1, 2, 3 or 4 h in the absence (Control, blue) or presence of cycloheximide (CHX, black). **A.** Nascent proteins visualised by confocal microscopy. Note the steady increase in signal in Control compared to animals lacking protein synthesis. Box schematizes the timeline of the experimental procedures. **B,C.** Quantification of OPP-PA fluorescence of somite 17 (n=4 fish at each timepoint). Signals were normalised to mean Control samples at 1 h (B), and compared after subtraction of CHX background (C). Bars represent mean ± SEM.

Figure S5

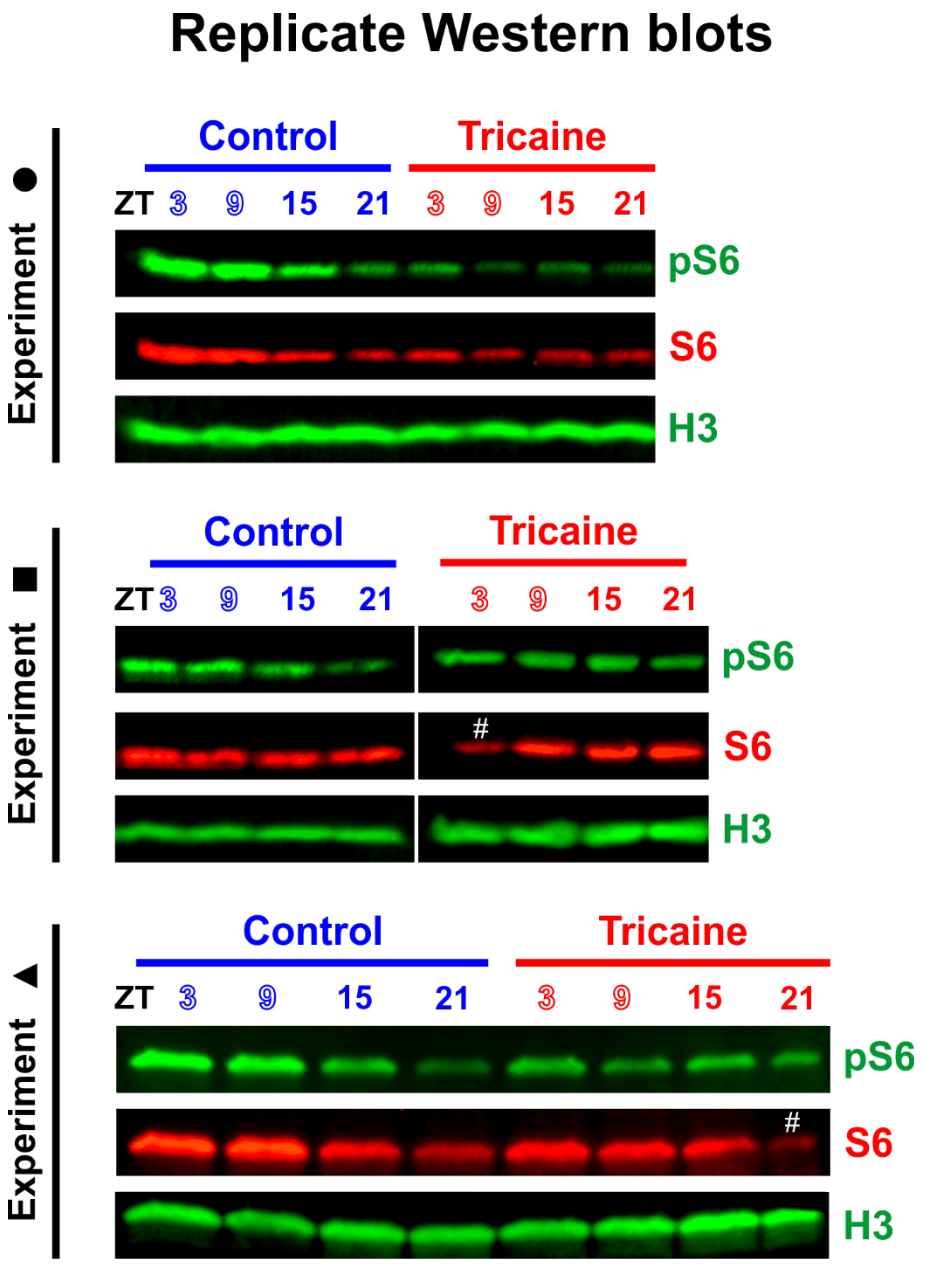

Fig. S5. Diurnal activity regulates S6 phosphorylation. Detection of pS6, total S6 and loading control H3 in replicate Western blots (including that shown in Fig. 3A) from three separate lays (shape symbols correspond to data presented in Fig. 3B) of fish grown in fish water (Control) or fish water plus Tricaine, consistently showing a) increased S6 at ZT3 in control, b) decreasing pS6/S6 ratio in control at ZT21 and c) ablation of daytime pS6/S6 increase by tricaine. Note that the two highest pS6/S6 points in tricaine (indicated by # and quantified in Fig. 3B) have unusually weak S6 bands rather than high pS6 bands, possibly indicating an S6 detection problem. Data in Experiment ■ are from two gels run and blotted in parallel.

Figure S6

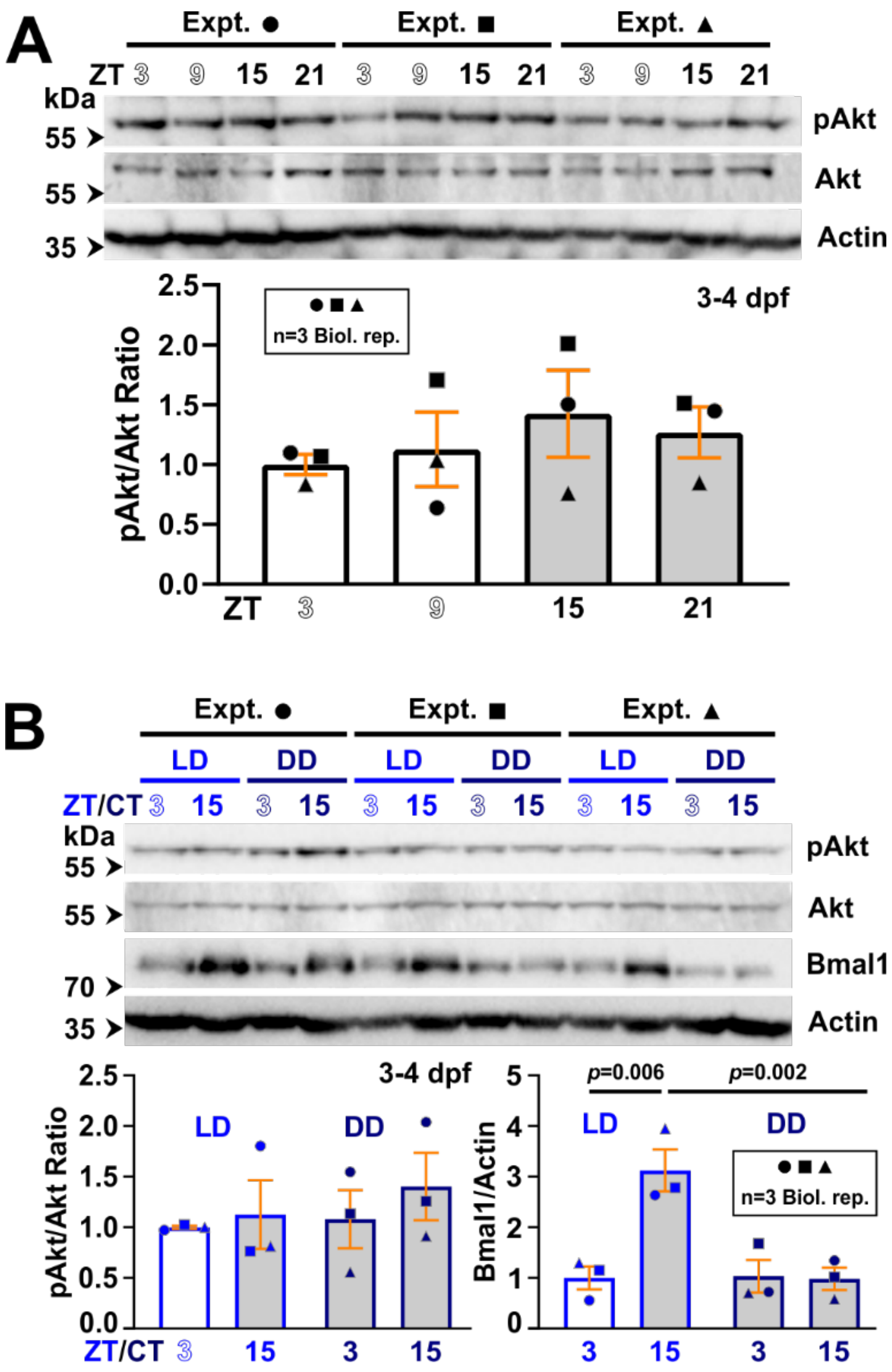

Fig. S6. Diurnal activity does not affect Akt phosphorylation. Immunodetection of pAkt, total Akt, Bmal1 and loading control actin. **A.** Replicate blots (on the same sample set shown in Figs 3A and S5) from three separate lays (shape symbols correspond to data presented in Fig. 3B) of fish grown in fish water showing no significant increase in daytime pAkt/Akt ratio. Ratios were normalised to LD samples at ZT3. **B.** Comparison of pAkt, Akt and Bmal1 protein level in LD and DD fish. Note the loss of cycling in Bmal1 under DD, but the lack of change in pAkt or Akt level. Ratios were normalised to LD samples at ZT/CT3.

Figure S7

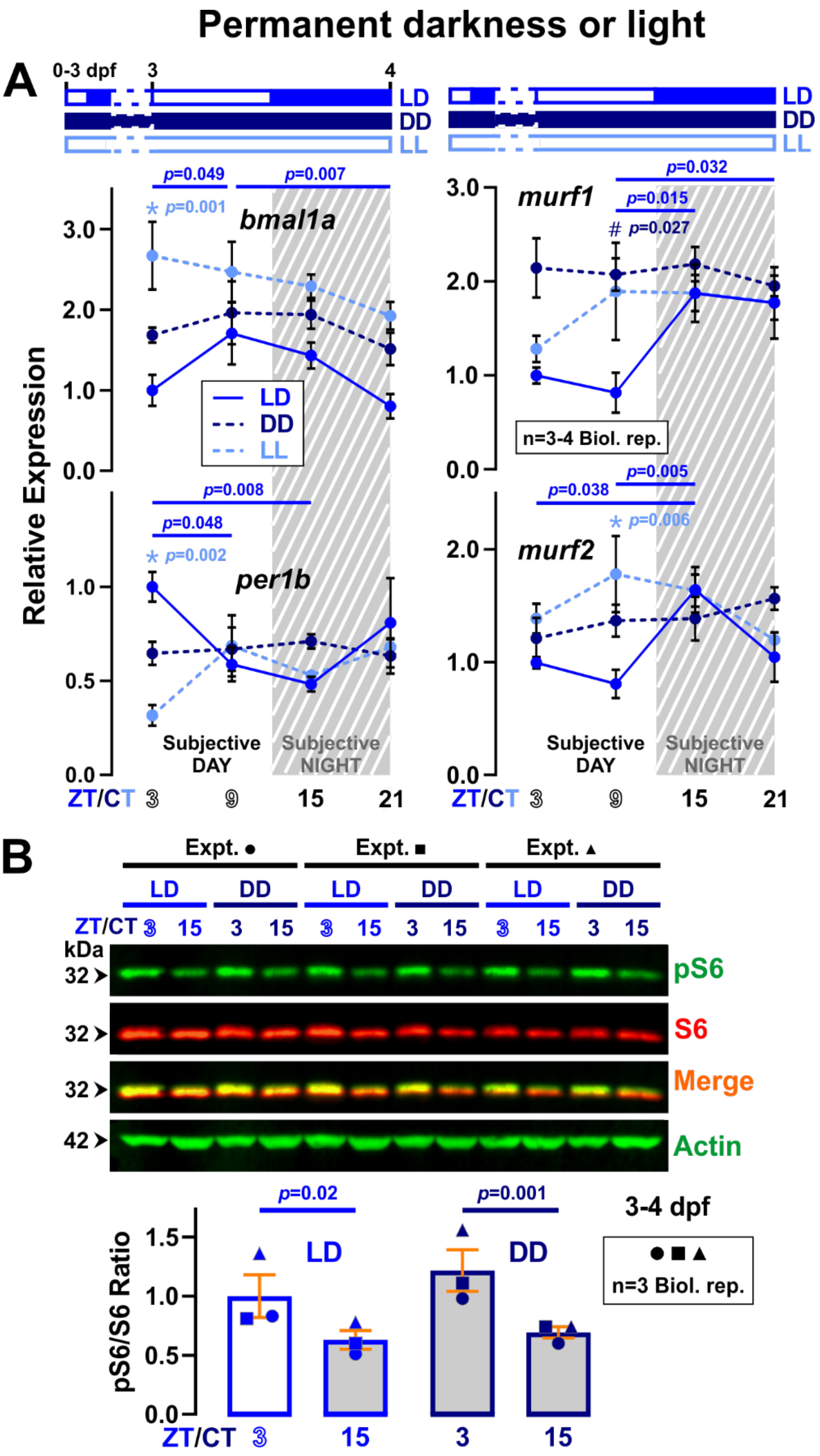

Fig. S7. Effect of permanent darkness or light on circadian entrainment and protein metabolism. A. Expression of the clock gene *bmal1a*, *per1b*, and atrophy-related genes *murf1* and *murf2,* was assayed by qPCR every 6 h between 3 and 4 dpf in larvae raised under LD (blue), DD (dark blue), or LL (light blue) from

0-4 dpf (n=3-4 biological replicates). *ef1a* was used for normalisation. # and * indicate significant differences between LD and DD, and between LD and LL, respectively. **B.** Western analysis of pS6, total S6, and actin loading control at ZT/CT3 and ZT/CT15 between 3 and 4 dpf in larvae raised under LD or DD. Same sample set with corresponding shape symbols shown in Fig. S6B. pS6/S6 ratios were normalised to LD samples at ZT3.

Figure S8

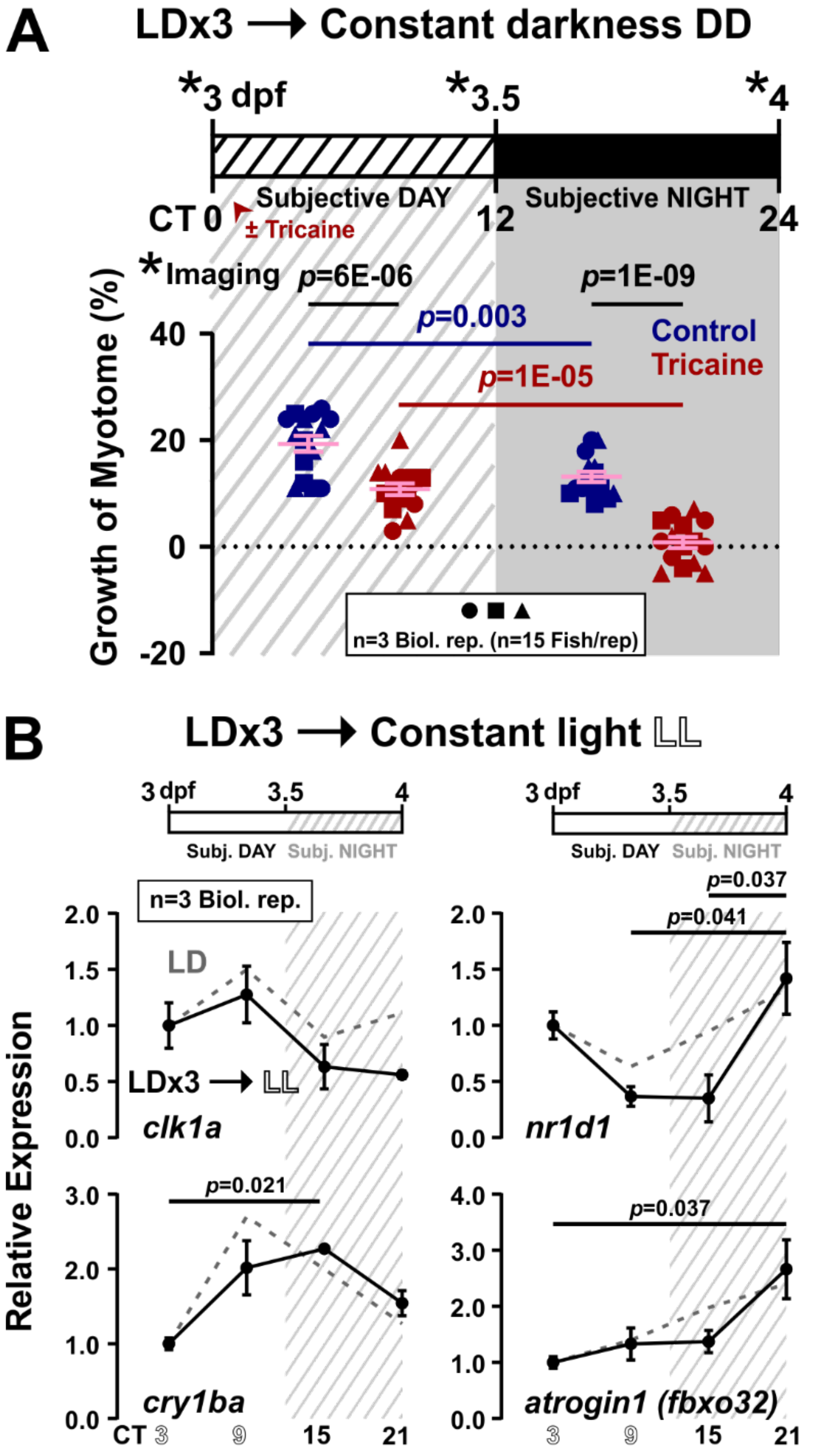

Fig. S8. Effect of short-term constant darkness or light on myotome growth and protein metabolism. **A.** Growth of myotome (%) in larvae raised under 3 cycles of LD and then switched to DD between 3 and 4 dpf (LD→DD) that were either unanaesthetised (Control, dark blue) or anaesthetised from 3-4 dpf (Tricaine, dark red). Colours represent different treatments. Symbols represent three distinct biological replicate lays (n=15 fish). **B.** Persistent circadian variation in the expression of clock genes, *clk1a*, *cry1ba*, and *nr1d1*, and atrophy-related gene *atrogin1* (*fbxo32*), between 3 and 4 dpf in larvae raised under LD→LL (n=3 biological replicates; black solid lines). *ef1a* was used for normalisation. Representative traces from LD fish in separate experiments are shown as references (grey dotted lines).

Figure S9

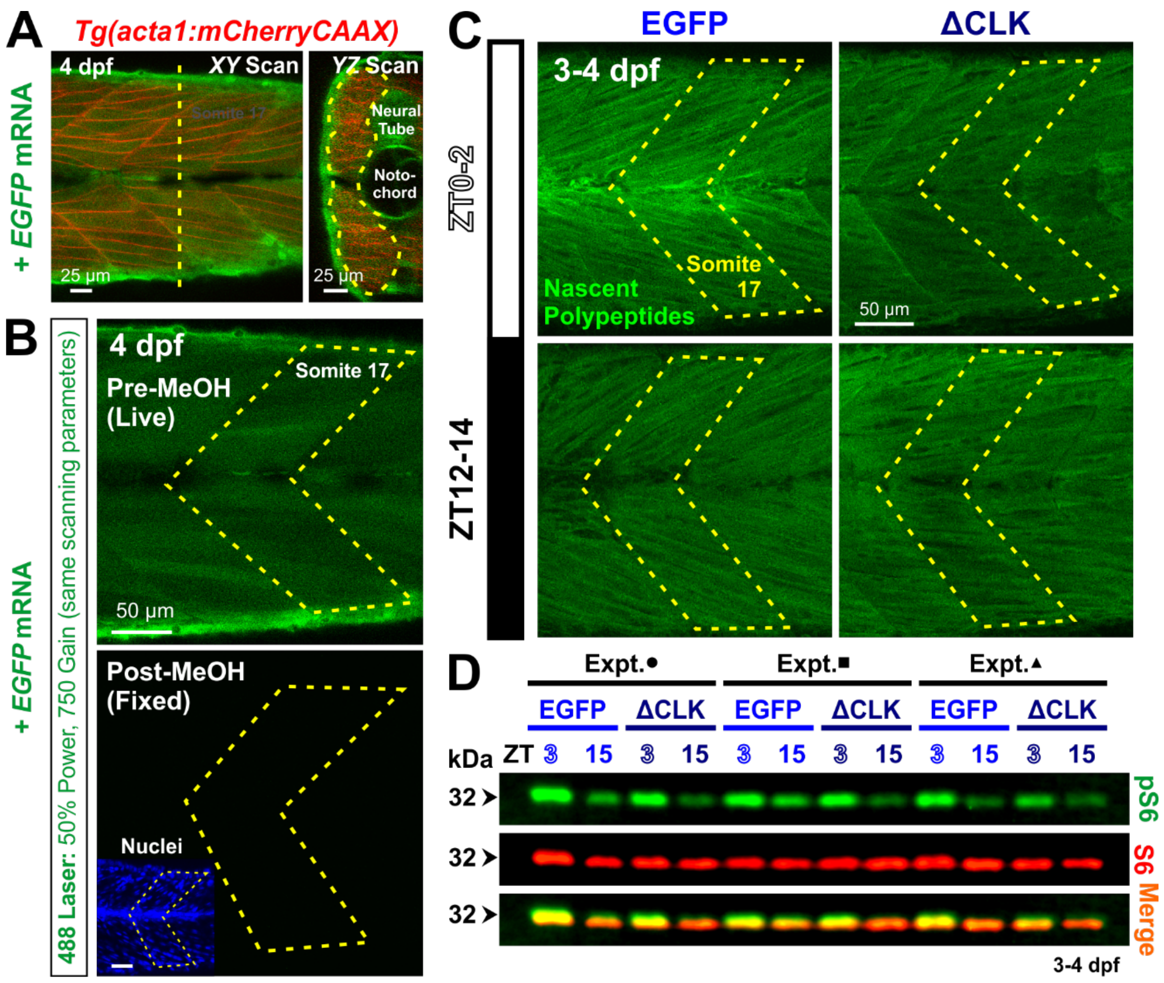

Fig. S9. Live imaging of *α-actin:mCherryCAAX* transgenic larvae, OPP incorporation and mTOR signalling after clock inhibition. Control and ΔCLK larvae were raised under LD and analysed at the time indicated between 3 and 4 dpf, shown in left lateral view (dorsal to top). **A.** Representative confocal images showing *XY* and *YZ* optical sections of somite 17 of a 4 dpf *α-actin:mCherryCAAX* transgenic fish injected with *EGFP* mRNA. **B.** Effective quenching of EGFP signal by MeOH allows visualisation of OPP-PA fluorescence in (C). Inset shows Hoechst 33342-stained nuclei. **C.** Nascent proteins visualised by OPP incorporation at ZT0-2 or ZT12-14 between 3 and 4 dpf showing greater incorporation during the day and its reduction by ΔCLK (quantified in Fig. 7A). **D.** Western analysis of pS6 and total S6 at ZT3 and ZT15 (Experiment shape symbols correspond to data presented in Figs 6B and 7B).

Table S1. Primers used for qPCR and cloning.

qPCR

Gene	NCBI ID	Sequence	Source
bmal1a	NM_131577.1	F: 5'- AGGGAAACGGCCTCTTCA-3'	(8)
		R: 5'- GCGTGGCAGTGATGTTTAA-3'	
clk1a	NM_130957.2	F: 5'-GCTGCAGTTTTCCACACAGA-3'	(8)
		R: 5'-ACCCTGTCCTTGAACCCTCT-3'	
cry1ba	NM_001313822.1	F: 5'-TCTACCAACAACTGTCCCGCTAC-3'	(9)
		R: 5'-GCCATCCCATTTCCATTCCC-3'	
ef1a	NM_131263.1	F: 5'-AGCAGCAGCTGAGGAGTGAT-'3	(10)
		R: 5'-CCGCATTTGTAGATCAGATGG-'3	
fbxo32 (atrogin1)	NM_200917.1	F: 5'-CTACATGAACATTCTGGAAAGG-3'	KiCqStart® SYBR® Green Primers (Sigma Aldrich)
		R: 5'-TGCAGAAGTTCTTTAATCGG-3'	
nr1d1	NM_205729.2	F: 5'-GAAGGCTGGAACATTTGAGGTC-'3	(11)
		R: 5'-GCAGACACCAGGACGACCG-'3	
per1b	NM_212439.2	F: 5'-ATCCAGACCCCAATACAAC-'3	(8)
		R: 5'-GGGAGACTCTGCTCCTTCT-'3	
murf1 (trim63a)	NM_001002133.1	F: 5'-TTCCGATGCCCTACTTGTCG-3'	(12)
		R: 5'-TTTCAAAGGGGGCTCAAGGG-3'	
murf2 (trim55b)	NM_001039982.1	F: 5'-CAGTATTTACCAGACGCAAA-3'	KiCqStart® SYBR® Green Primers (Sigma Aldrich)
		R: 5'-ACCATCATTGCTATTCCATC-3'	

Molecular cloning

Cloning	Sequence	Note
EGFP-2A-ΔCLK-5xMyc	F: 5'-CTCGAGCCTCTAGAGCCACCATGGTGAGCAAGGGCGAG-3'	For cloning of the *EGFP-2A-ΔCLK-5xMyc* from the *pT2-aanat2-EGPF-2A-ΔCLK-5xMyc*.
	R: 5'-ATCATGTCTGGATCTACGTATCACATGGTGAGGTCGCC-3'	
pCS backbone	F: 5'-TACGTAGATCCAGACATGATAAGATACATTG-3'	For removing the *mCherryCAAX* fragment from the *pCS-mCherryCAAX*.
	R: 5'-GGTGGCTCTAGAGGCTCG-3'	

Supplementary Information References

1. T. K. Tamai, L. C. Young, D. Whitmore, Light signaling to the zebrafish circadian clock by Cryptochrome 1a. *Proc. Natl. Acad. Sci. U. S. A.* **104**, 14712–14717 (2007).

2. M. P. S. Dekens, D. Whitmore, Autonomous onset of the circadian clock in the zebrafish embryo. *EMBO J.* **27**, 2757–2765 (2008).

3. K. Lahiri, *et al.*, Temperature regulates transcription in the zebrafish circadian clock. *PLoS Biol.* **3**, e351 (2005).

4. R. Diogo, Y. Hinits, S. M. Hughes, Development of mandibular, hyoid and hypobranchial muscles in the zebrafish: homologies and evolution of these muscles within bony fishes and tetrapods. *BMC Dev. Biol.* **8**, 24 (2008).

5. K. J. Livak, T. D. Schmittgen, Analysis of relative gene expression data using real-time quantitative PCR and the 2(-Delta Delta C(T)) Method. *Methods* **25**, 402–408 (2001).

6. Z. B.-M. Livne, *et al.*, Genetically Blocking the Zebrafish Pineal Clock Affects Circadian Behavior. *PLOS Genet.* **12**, e1006445 (2016).

7. M. E. Hughes, J. B. Hogenesch, K. Kornacker, JTK_CYCLE: an efficient nonparametric algorithm for detecting rhythmic components in genome-scale data sets. *J. Biol. Rhythms* **25**, 372–380 (2010).

8. N. Hamilton, N. Diaz-de-Cerio, D. Whitmore, Impaired light detection of the circadian clock in a zebrafish melanoma model. *Cell Cycle* **14**, 1232–1241 (2015).

9. M. Wang, Z. Zhong, Y. Zhong, W. Zhang, H. Wang, The zebrafish period2 protein positively regulates the circadian clock through mediation of retinoic acid receptor (RAR)-related orphan receptor α (Rorα). *J. Biol. Chem.* **290**, 4367–4382 (2015).

10. J. J. Kelu, S. E. Webb, A. Galione, A. L. Miller, Characterization of ADP-ribosyl cyclase 1-like (ARC1-like) activity and NAADP signaling during slow muscle cell development in zebrafish embryos. *Dev. Biol.* **445**, 211–225 (2019).

11. H. Wang, *et al.*, Single-cell in vivo imaging of cellular circadian oscillators in zebrafish. *PLOS Biol.* **18**, e3000435 (2020).

12. M. Li, M. Andersson-Lendahl, T. Sejersen, A. Arner, Knockdown of fast skeletal myosin-binding protein C in zebrafish results in a severe skeletal myopathy. *J. Gen. Physiol.* **147**, 309–322 (2016).
